# Acquisition-invariant brain MRI segmentation with informative uncertainties

**DOI:** 10.1016/j.media.2023.103058

**Published:** 2023-12-07

**Authors:** Pedro Borges, Richard Shaw, Thomas Varsavsky, Kerstin Klaser, David Thomas, Ivana Drobnjak, Sebastien Ourselin, M. Jorge Cardoso

**Affiliations:** a Department of Medical Physics and Biomedical Engineering, UCL, UK; b School of Biomedical Engineering and Imaging Sciences, KCL; c Dementia Research Centre, UCL, UK

**Keywords:** MRI physics, Harmonisation, Deep Learning, Simulation, Uncertainty modelling

## Abstract

Combining multi-site data can strengthen and uncover trends, but is a task that is marred by the influence of site-specific covariates that can bias the data and, therefore, any downstream analyses. Post-hoc multi-site correction methods exist but have strong assumptions that often do not hold in real-world scenarios. Algorithms should be designed in a way that can account for site-specific effects, such as those that arise from sequence parameter choices, and in instances where generalisation fails, should be able to identify such a failure by means of explicit uncertainty modelling. This body of work showcases such an algorithm that can become robust to the physics of acquisition in the context of segmentation tasks while simultaneously modelling uncertainty. We demonstrate that our method not only generalises to complete holdout datasets, preserving segmentation quality but does so while also accounting for site-specific sequence choices, which also allows it to perform as a harmonisation tool.

## 1 Introduction

The substantial soft-tissue contrast of MRI makes it the tool of choice in a myriad of applications, especially in the field of neuroimaging. The largely non-quantitative nature of MRI means that information is derived from the relative contrast between tissues rather than the value of the signal in said tissues. Different sequence and sequence parameter choices will result in the emphasis of different tissues, and these are deliberately chosen depending on the task at hand (Chavhan, 2016). There is therefore a high demand for algorithms that can suitably handle such contrast varying data. Such methods should ideally not only be able to perform adequately on data arising from multiple sites and/ or acquired using multiple protocols, but they should also do so while directly accounting for the biases that arise from these variations. This is known as harmonisation and allows comparisons to be drawn as if all data had been acquired in the same fashion from a single site.

## 2 Related works

### 2.1 Improving model generalisability

Many techniques have been proposed for improving generalisability. Probabilistic generative models (Ashburner and Friston, 2005) are widespread in their use but are limited by their strict label intensity distributions and underlying assumptions. Multi-atlas fusion methods (Sabuncu et al., 2010) are likewise popular but suffer from prolonged processing times owing to their registration-based nature.

Convolutional neural networks (CNNs) have achieved state-of-the-art results in a multiplicity of medical imaging tasks including image-to-image translation, segmentation, and classification (Yu et al., 2021). CNNs, however, are susceptible to overfitting to the data regime in which they were trained and evaluated, resulting in poor generalising performance, and limiting their use for clinical applications (Yasaka and Abe, 2018)(Karani et al., 2018). This is due to the fact that the data used for training may not exhibit overlapping characteristics with the holdout set, the set of data that may have arisen, for example, from a different site that may have employed a different scanning protocol. This can be ameliorated with a robust augmentation scheme, but standard augmentation pipelines cannot replicate contrast differences between regions without further modelling, leading to inconsistent biomarker extraction (Shinohara et al., 2017).

Ideally, CNNs would be trained on contrast-rich, manually annotated, datasets to maximise their generalizability, as this exposes them to a wide variety of visual features and patterns which should help them to develop robust and flexible representations of the data, making them more resilient to the variability that arises from, for example, poor contrast, noise, or artifacts. In practice however such datasets are scarce, owing to the difficulty in aggregating multi-site data and to how time-consuming and costly it can be to obtain annotations (Hosny et al., 2018). As a result, we must look towards methodological improvements that can tackle this contrast consistency conundrum.

Zhang et al. (2020) train networks with a robust augmentation scheme that includes image sharpening, blurring, the addition of noise, brightness and contrast adjustments, affine transforms, and free-form deformation. They show that models trained in this fashion are more able to aptly generalise to unseen domains, owing to their augmentation scheme capturing most potential sources of image variability.

Pham et al. (2020) designed a method whereby contrast robustness is attained by training a segmentation-synthesis model. A standard segmentation network is trained, which is then used to segment an image of unseen contrast. This (initially suboptimal) segmentation is then used to train a synthesis network that is applied in combination with the observed contrast images’ labels to regenerate these contrast images, which in turn serve to retrain the segmentation network, and the process is repeated. By sampling intensities from Gaussian mixture models for different regions in ATLAS sampled label maps, images of unrealistic, but extreme contrast variations can be generated, which forms the basis for SynthSeg (Billot et al., 2021). When paired with a robust augmentation scheme very similar to that of Zhang et al. (2020), it is shown that this methodology generalises well to images of different modalities without requiring any fine-tuning. PSACNN (Jog et al., 2019) leverages approximate static equation models in conjunction with pseudo-physics parameter estimation to generate synthetic images which are used to train networks that will generalise to a target dataset. Varadarajan et al. (2021) propose a means of generating quantitative MR maps using a small set of multi-echo FLASH images in a manner that is robust to the input acquisition parameters. Crucially, these generalisability-related methods do not explicitly account for the biases introduced by the choice of imaging parameters. To elaborate, what all these generalisability methods lack is the ability to explicitly account for the biases (e.g.: contrast variations) that are introduced as a result of variables relating to the physics of image acquisition, namely the choice of sequence and sequence parameters. Such models will perform their task according to the visual properties of the image alone, as they do not have any accompanying information relating to the acquisition, nor are they trained in a fashion that might convey this information implicitly. This leads to a discussion on data harmonisation.

### 2.2 Harmonisation

It is important to acknowledge works that approach the problem of multi-site harmonisation. For example, ComBat is a Bayesian method that models site effects both additively and multiplicatively, allowing data harmonisation while preserving biological variability (Johnson et al., 2007). Harmonisation has also been taken on with CycleGANs (Zhu et al., 2017; Zhao et al., 2019) and domain adaptation approaches (Dinsdale et al., 2020).

Even when designing models that exhibit greater generalisability, there will still be instances of poorer performance, or failure. Incorporating uncertainty estimation into our algorithms allows for an additional degree of insight and safety to be attached to every prediction, and because of these boons, it has featured increasingly in deep learning works, including medical imaging (Klaser et al., 2021) (Tanno et al., 2021) (Graham et al., 2020) (Horner et al.). Acquisition parameters change tissue contrast and noise, to such an extent where certain choices of parameters can result in very uncertain segmentations, due to the lack of contrast. We can therefore leverage uncertainty estimation to measure the model’s ability to perform a task as a function of the acquisition parameters. We choose to model both epistemic and heteroscedastic aleatoric uncertainty (Kendall and Gal, 2017). Epistemic uncertainty relates to the uncertainty in the model, while heteroscedastic aleatoric uncertainty relates to the uncertainty intrinsic to the data.

In our previous work (Borges et al., 2020), we proposed a method that explicitly models the physics of the acquisition to design networks that can become invariant to the process by which they were acquired. This method made use of multiparametric MR maps (MPMs), which contain voxelwise quantitative MR parameter information combined with MR sequence simulations to generate images of various contrasts which are used to train networks privy to the sequence parameters used to generate the synthetic images. We also put forward a *Physics Gold Standard* (PGS) label creation model to generate the tissue segmentations used to train these networks. We described the PGS as a true anatomical ground truth, as it is derived directly from the quantitative MPMs, and therefore not affected by the biases that a segmentation created from a qualitative image would have introduced. The work showed that networks trained in this fashion can achieve greater segmentation consistencies across a wide range of sequence parameters without incurring detriments to the segmentation quality. This work did not, however, offer out-of-distribution analyses, nor was this work validated extensively on real, external datasets.

### 2.3 Contributions

In this work, we address these limitations and introduce several improvements that significantly contribute towards acquisition-invariance, namely, the translation of the proposed simulation framework into a full dynamic data augmentation pipeline, the introduction of a contrast stratification loss, and the modelling of uncertainty. We hypothesise that these modifications should allow for a greater extrication of the physics of the acquisition and the underlying anatomy and a greater ability to extrapolate to unknown regions of contrast and parameter space. This is evaluated by means of analysing segmentation consistency across in and out of distribution samples within subjects, as well as how the uncertainty-derived volumetric uncertainties differ between methods. Furthermore, we investigate how our method performs on a harmonisation task consisting of real multi-site data by analysing the consistency of predicted longitudinal tissue trends across sites.

With this work, we sought to design a method that can (1) generalise aptly to varying contrasts while (2) implicitly harmonising results (due to its knowledge of the physics of the acquisition process) and (3) provide uncertainty quantification that can be directly tied to the choice of protocol parameters. Methods such as PSACNN and SynthSeg address the problem of generalisability without explicitly addressing harmonisation. The various outlined harmonisation methods either harmonise post-hoc (ComBat) or do so in a fashion that is specific to the data at hand (Zhu et al., 2017; Zhao et al., 2019; Dinsdale et al., 2020) (i.e., The sources of variability are not generally modelled). Our method fulfils all three criteria and is thoroughly validated on real multi-protocol and multi-site data.

## 3 Methods

### 3.1 Framework background

In our original work (Borges et al., 2020), we proposed and implemented an implicitly harmonising physics-informed segmentation framework trained with synthetic data. Images were generated using a simple static-equation-based physics forward model that employed MPMs. Our labels, which we referred to as a *Physics Gold Standard* (PGS) were created by sourcing literature values for the *R*_1_ values for the tissues of interest, grey matter (GM), white matter (WM), and cerebrospinal fluid (CSF) and fitting a Gaussian mixture model for each on the quantitative values in the MPMs, the very same ones used to generate our images, thus giving us paired data to train with. Our architecture of choice was a modified U-Net, which, alongside our simulated images, accepted the physics parameters used to generate said images into a separate branch that merged into the first and penultimate set of convolutional layers. We showed that while the network boasted harmonisation abilities in some instances and was able to perform well in a databridging setting, it disappointingly did not always out-compete a complete baseline.

### 3.2 Major methodological changes overview

Having outlined the foundational method, we summarise the proposed methodological additions as follows:

**Simulation augmentation**: By casting the simulation pipeline as an augmentation layer rather than keeping it as a pre-processing step, it allows for images to be generated during training on demand, with a greater exploration of contrast space.
**Batch stratification and loss**: By simulating multiple contrasts for a single subject (who will have a single, consistent PGS ground truth), combining them in a batch, and enforcing that network features for all contrasts are as close as possible by incorporating a regression-type stratification loss the network is given an explicit harmonisation objective during training.
**Uncertainty quantification**: By incorporating both epistemic and aleatoric uncertainty modelling into the network and distilling volumetric bounds for output segmentations, an additional level of safety and insight is granted to the model.

### 3.3 Section summary and introduction

We cover in this section the various methodological improvements to the physics-informed segmentation method introduced in our original work (Borges et al., 2020).

We begin by covering the architecture under *Network architecture*, followed by the introduction of changes to the original training paradigm, featuring a new loss in *Stratification and batch homogeneity*; changes to the simulation portion of our pipeline in *Casting simulation as an augmentation layer*, and lastly a delineation of uncertainty modelling and how we incorporate it into our training and validation scheme features in *Uncertainty modelling*.

As in our previous work (Borges et al., 2020), we continue using the PGS, by sourcing literature values for the *R*_1_ values for the tissues of interest, grey matter (GM), white matter (WM), and cerebrospinal fluid (CSF) and fitting a Gaussian mixture model for each on the quantitative values in the MPMs. Namely, the normal distribution for GM is defined as 𝒩(0.683, 0.080^2^), for WM as 𝒩(1.036, 0.080^2^) (Weiskopf et al., 2013), and for CSF as 𝒩(0.240, 0.030^2^) (Rooney et al., 2007), in units of seconds.

The *Physics Gold Standard* exhibits interesting properties typically absent from manual annotations. Firstly, in assigning voxels to tissues according to a GMM, the resultant labels are not categorical and more accurately reflect the continuous nature of tissue boundaries. In the same vein, certain structures, such as the Thalamus, Amygdala, Caudate Nucleus (which cannot be fully labelled as grey matter), and the motor cortex (which is fairly myelinated compared to other cortical areas) more obviously have this property reflected in our *Physics Gold Standard* maps, which again would go unnoticed in categorical labelling.

All volumes are skull-stripped to ensure no further extracranial tissue considerations are required. For this, we employed Geodesic Information Flows (GIF) (Cardoso et al., 2015), treating the total internal volume outputs (TIV) as an intracranial mask. GIF requires *T*_1_ contrasted images, and to this end, we simulated one MPRAGE image for each subject with a TI of 900ms (A value within the theoretical optimal range Wang et al. (2014)) in order to obtain intracranial masks for all subjects. There will be a bias towards the type of contrast chosen to obtain the intracranial mask. However, in practice, we verify that the difference between masks across a wide inversion time range is small, namely less than 0.5% total volume difference across a 600 to 1200 ms inversion time simulation range.

We propose improvements to our previous methodology, further validating our method on out-of-distribution samples aided by uncertainty-derived errors and evaluating our method on a multi-site harmonisation task to assess generalisability to a real, multi-contrast dataset.

### 3.4 Network architecture

We adopt the nn-UNet architecture (Isensee et al., 2018) owing to its widespread adoption for segmentation tasks in literature.

**Fig. 1:**
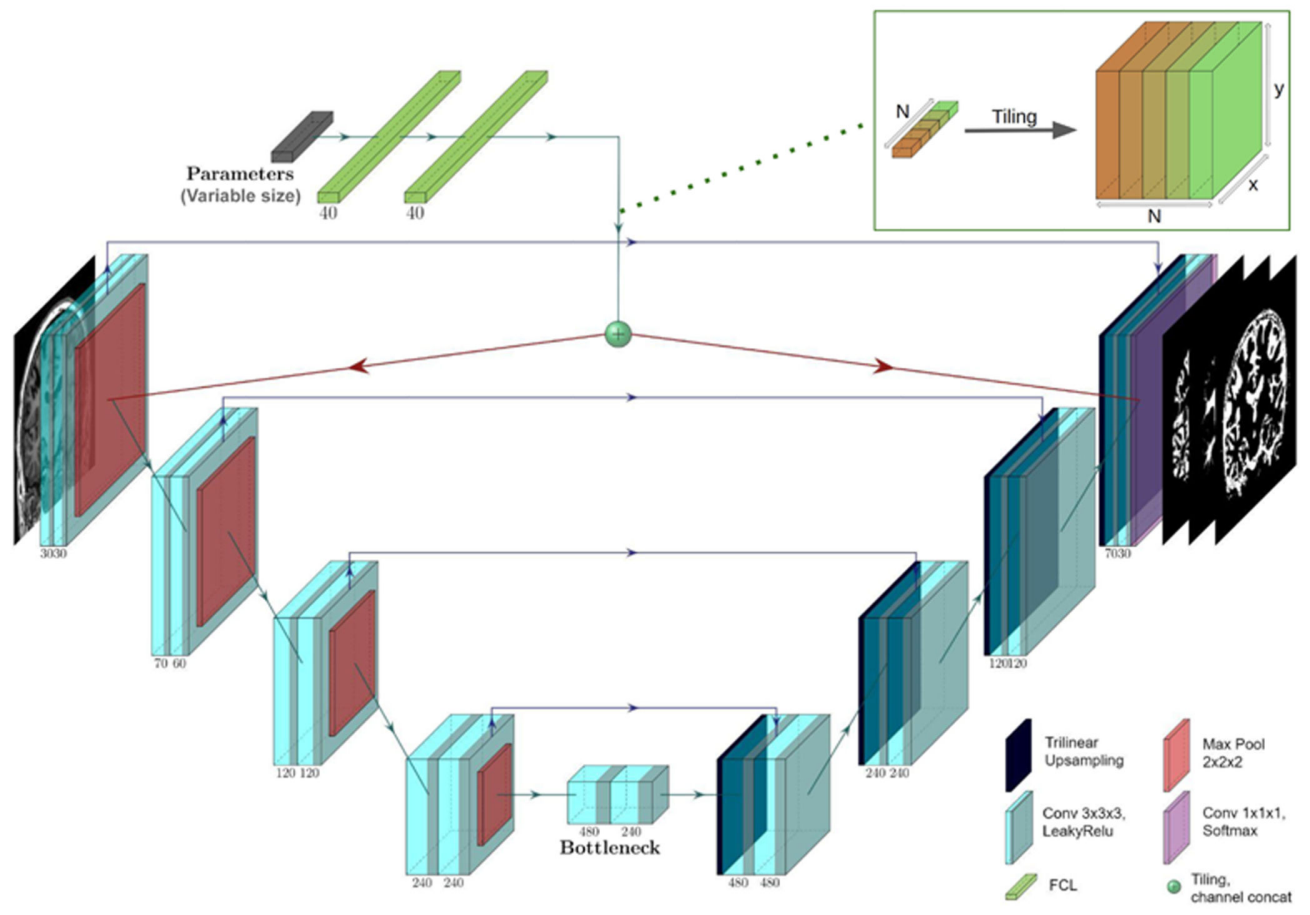
The 3D physics-informed architecture. The numbers below the blocks denote the number of channels in the feature maps following the convolution it is associated with. The output features of the fully connected layers are tiled to be compatible in size with the main network convolutional features they are concatenated to, as shown in the top right.

The network consists of four contracting blocks, a bottleneck layer, four expansive blocks, and an output block. Each contracting block consists of a kernel size three 3D convolution and leakyrelu activation pairs. The bottleneck layer and expansive blocks are each made up of the same pair of operations followed by a non-parametric trilinear upsampling layer; see Figure 1.

As in our original work, the acquisition parameters used to generate the images in any one batch are passed via two fully connected layers before tiling the output of said layers, both following the second convolutional layer and following the second to last convolutional layer. We posit that knowledge of the physics can enrich the features learned in the encoding part of the network instead of constraining the physics knowledge to the end layers of the network alone.

MONAI (Consortium”, 2020), TorchIO (Pérez-García et al., 2020), and PyTorch are used for all implementations.

### 3.5 Stratification and batch homogeneity

Intra-subject segmentation volume consistencies across multiple contrasts can be considered as a surrogate for acquisition parameter invariance. We propose some changes to the original methodology to further enforce this consistency. In particular, we leverage the fact that, for a single subject, the PGS segmentations are constant for every simulated realisation of the images (regardless of the choice of sequence and sequence parameters used to simulate images for that subject), as the underlying biology is unchanged. If a batch only contains realisations from a single subject, and the patch location is identical for all samples in the batch, then the labels for this batch are also identical. If using single subject batches, we can therefore add a constraint to the batch feature maps to enforce similarity between them. This comes in the form of a stratification (*L*_2_) loss (equation 1 below) over all the feature maps in the penultimate layer of the network, which is added to the standard cross-entropy loss to form our final proposed loss function (equation 2 below):

(1) $$L_{Stratification\;}=-\frac{1}{C_{2}^{n}}\sum_{\begin{array}{c} a=1,b=2\\ a\neq b \end{array}}^{n}{\left(F_{a}-F_{b}\right)}^{2}$$

(2) $$L_{Total\;}=-\sum_{i=1}^{n}c_{i}\text{log}\left(p_{i}\right)-\frac{1}{C_{2}^{n}}\sum_{\begin{array}{c} a=1,b=2\\ a\neq b \end{array}}^{n}{\left(F_{a}-F_{b}\right)}^{2}$$

Where $C_{2}^{n}$ is the binomial coefficient, denoting the total number of unique pairs of combinations of feature maps in the batch, ${\text{Σ}}_{a\neq b}^{a=1,b=2^{n}}$ represents a sum over all possible unique pairs, and *F* denotes a single feature map. E.g. For three feature maps (A, B, and C), an *L*_2_ is calculated between feature maps A and B, feature maps A and C, and feature maps B and C, where each of these pairings refers to one such unique combination (Note that feature maps are never compared against themselves).

The first term of the second equation denotes the standard cross-entropy loss, with *c*_*i*_ as the voxelwise ground truth label and *p*_*i*_ as the probabilistic voxelwise network prediction (i.e., the Softmax of the output network logits).

We term this loss *stratification* because its inclusion forces features to be the same, therefore stratifying style and content.

### 3.6 Casting simulation as an augmentation layer

The static equation simulation approach follows that described in Jog et al. (2015); specifically, we make use of the MPRAGE and SPGR equations described there. These approximate the signal, per voxel, given its intrinsic MR parameters (*T*_1_, $T_{2}^{*}$, *PD*) and the chosen sequence parameters, which depend on the specific sequence being modelled. The absence of a temporal component is why they are termed static. The static signal for voxel *x* for an MPRAGE sequence is:

(3) $$b_{M}(x)=G_{S}PD(x)\left(1-\frac{2e^{\frac{-TI}{T_{1}(x)}}}{1+e^{\frac{-(TI+TD+\tau )}{T_{1}(x)}}}\right),$$

Accordingly, for an SPGR sequence:

(4) $$b_{S}(x)=G_{S}PD(x)sin\theta \frac{1-e^{-\frac{TR}{T_{1}(x)}}}{1-\text{cos }\theta e^{-\frac{TR}{T_{1}(x)}}}e^{-\frac{TE}{T_{2}^{*}(x)}},$$

For the sequence-specific parameters, *G*_*S*_ denotes the scanner gain, *T I* is time between the inversion recovery pulse and the first RF readout pulse, *TR* the repetition time, *TE* the echo time, τ the echo spacing time, *TD* the delay time, and θ the flip angle. *G*_*S*_ is a multiplicative factor that is assumed to be constant for all voxels, so is chosen to remain constant for all simulations. Note that the proposed static equation model is an approximation of the imaging process which ignores the local MRI dynamics, but allows for sufficiently-realistic and fast simulations necessary for CNN model training.

In our earlier work, (Borges et al., 2020), the synthetic image creation process is part of pre-processing; a set number are pregenerated according to a set parameter interval and is then used for training. We propose casting the static equation simulation process as an augmentation. In this fashion, the network takes in a protocol type, a range of relevant protocol parameters sampled, and MPMs. Per iteration, a single MPM is selected, and by sampling from the range of protocol parameters N times, N simulated volumes are generated to make up the batch. This is in accordance with our aforementioned batch stratification modification, which therefore means that the selected patch for each of these samples resides in the exact same space. It is worth noting here that the augmentation layer feature does partially fall under the realm of data handling rather than being a purely methodological addition. Given that its inclusion is intrinsically tied to the passing of physics information and the batch stratification feature, however, we see it fit to include it under the same umbrella for the purposes of this work.

**Fig. 2:**
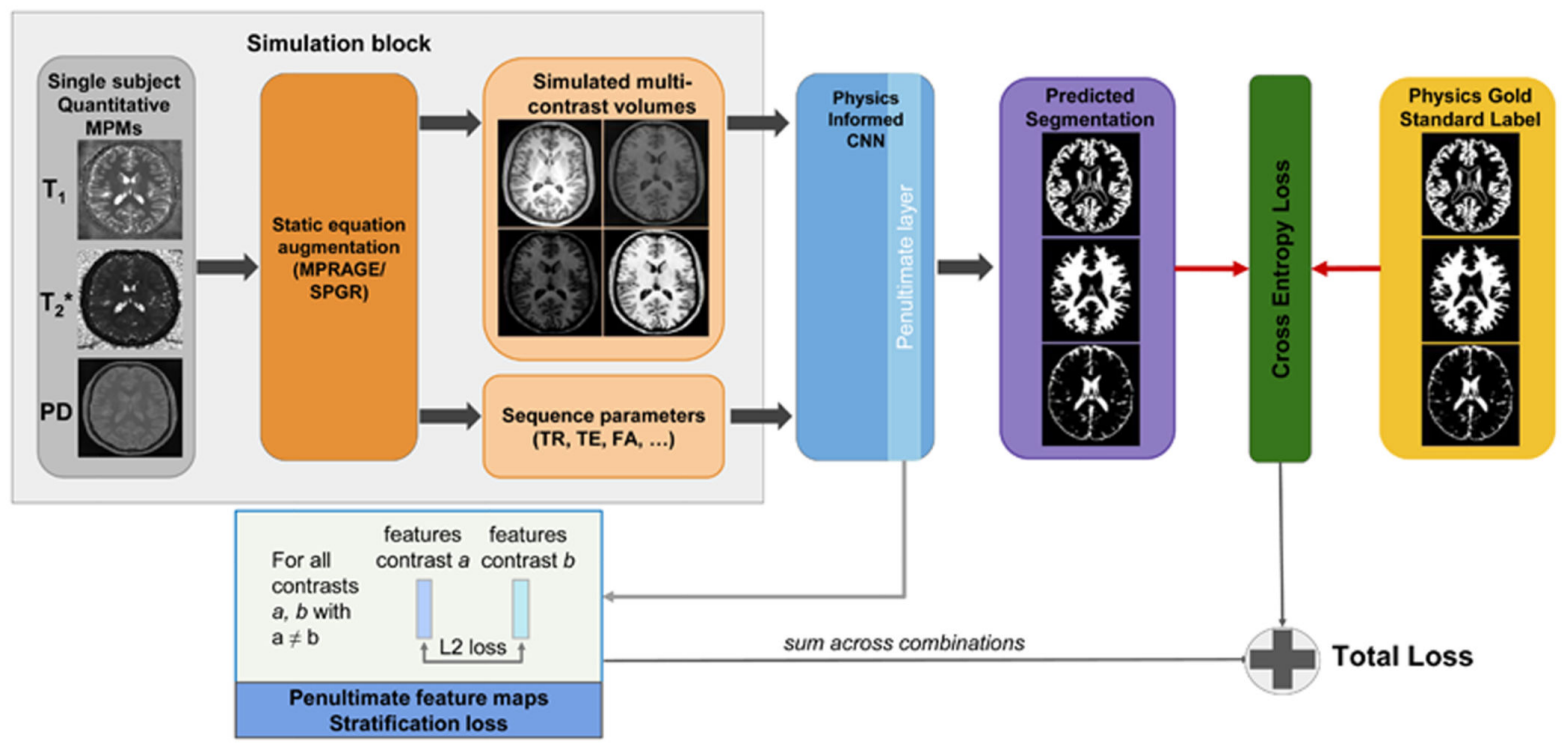
The training pipeline with proposed new additions of single subject batch stratification and accompanying *L*_2_ feature maps loss, and training time image simulation.

This eliminates the need for having to prepare the data in advance and allows for a greater dynamic exploration of the physics parameter space. Fig. 2 shows the training pipeline, featuring all aforementioned modifications.

In addition, we employ the standard MR-related training time data augmentations employed in the original work Borges et al. (2020), namely bias field and noise.

### 3.7 Uncertainty modelling

Data acquired with different parameters and devices results in differing levels of image contrast and noise, thus affecting a model’s ability to segment a target region of interest; the model’s uncertainty can characterise this effect. Modelling uncertainty allows us to obtain volumetric error bounds on our network outputs, which we can use to compare methods and model performance with in and out of distribution samples.

#### 3.7.1 Epistemic uncertainty

Epistemic uncertainty relates to model inadequacies, either due to limited data and/ or limited model capacity. For this reason, it is not uncommon to refer to it as a “knowable” uncertainty, as greater data availability and a more complex model serve to minimise epistemic uncertainty.

We choose test-time dropout with Monte Carlo sampling to model epistemic uncertainty. At training time, neurons are randomly ignored at some rate, *MC*_*r*_, every iteration. At test time, given any one input, multiple segmentations can be produced by maintaining this random neuron activation behaviour, essentially sampling from a series of sub-nets, which approximates Bayesian posterior sampling (Gal and Ghahramani, 2015).

For a given input, the epistemic uncertainty can then be determined by calculating the degree of variation that exists within its Monte Carlo segmentation samples. We are interested in obtaining a quantitative measure of the epistemic uncertainty, so we look at the variation in the segmentation volumes (obtained by summing the voxel values for each tissue class, per sample) rather than carrying out a voxel-wise analysis.

The dropout rate, *MC*_*r*_, is set to 50% in all layers to maximise the variance of those layers’ outputs, except for the first layer, where we opt for a value of 5% to not penalise the learning of low-level image features too harshly (Eaton-Rosen et al., 2018). No changes to the loss function are incurred as a result of employing Monte Carlo dropout.

#### 3.7.2 Aleatoric uncertainty

Aleatoric uncertainty relates to the irreducible noise present in the data, and does not decrease in the limit larger amounts of data, or greater modelling complexity. The heteroscedastic subcategory more specifically refers to aleatoric uncertainty that varies across inputs. MR images are not equally noisy, making heteroscedastic, rather than homoscedastic, aleatoric uncertainty the most appropriate to model. Note that noise in this instance does not solely refer to the random signal variations present in images, but also to blurry boundaries between tissues.

Heteroscedastic aleatoric uncertainty is explicitly modelled by means of loss attenuation as described by Kendall and Gal (2017). Our model incurs architectural and loss changes as a result of this. This new formulation begins by casting the network outputs as a linear sum between its task logits and noise sampled from a zero-meaned normal distribution with a standard deviation equal to voxelwise heteroscedastic uncertainty predictions. Propagating this to the cross-entropy loss, we arrive at a new formulation:

Let us begin by defining some starting variables. $f_{i}^{W}$ rep-resents the task logits (prior to Softmax) for the *i*th voxel. ϵ_*i*,*t*_ represents a sample taken from a zero-meaned normal distribution with a standard deviation $\sigma_{i}^{W}$

${\hat{\eta }}_{i,t}$ is the result of summing $f_{i}^{W}$, the voxelwise task logits, with samples taken from a normal distribution with mean zero and standard deviation equal to the voxelwise network prediction of $\sigma_{i}^{W}$.

${\hat{\eta }}_{i,t}$ is the result of summing $f_{i}^{W}$ to this stochastic sample ${\hat{\eta }}_{i,t}$. Because *η* is drawn randomly from a distribution, it is parameterised by *t*, which exists to showcase how this process is non-deterministic, as opposed to $f_{i}^{W}$, which (in a non-dropout setting) is, conversely, deterministic.

(5) $${\hat{\text{η}}}_{i,t}=f_{i}^{W}+\epsilon_{t},\;\epsilon_{t}∼N\left(0,{\left(\sigma_{i}^{W}\right)}^{2}\right)$$

Having defined these variables, we can move to define our loss, which is a simple Cross-Entropy loss taken over all voxels ***i*** and samples ***T. c***_***i***_ represents the ground truth voxelwise categorical labels, while ***SM*** is the Softmax operator.

(6) $$ℒ=-\underset{i}{\sum^{\text{​}}}\frac{1}{T}\underset{t}{\sum^{\text{​}}}c_{i}log\left(SM\left({\hat{\text{η}}}_{i,t}\right)\right)$$

By expanding the Softmax operation, we arrive at the following formulation, where, for the denominator of the final expression, the sum is taken over classes, denoted by ***c***^′^:

(7) $$ℒ=-\underset{i}{\sum^{\text{​}}}\frac{1}{T}\underset{t}{\sum^{\text{​}}}c_{i}log\left(\frac{e^{{\hat{\text{η}}}_{i,t}}}{{\text{Σ}}_{c\prime }e^{{\hat{\text{η}}}_{i,t,c\prime }}}\right)$$

Lastly, a few final rearrangements lead us to the final form of our loss:

(8) $$ℒ=-\underset{i}{\sum^{\text{​}}}\frac{1}{T}\underset{t}{\sum^{\text{​}}}c_{i}\left(log\left(e^{{\hat{\text{η}}}_{i,t}}\right)-log\left(\underset{c^{\prime }}{\sum^{\text{​}}}e^{{\hat{\eta }}_{i,t,c^{\prime }}}\right)\right)$$

(9) $$ℒ=\underset{i}{\sum^{\text{​}}}log\frac{1}{T}\underset{t}{\sum^{\text{​}}}exp\left(c_{i}\left(-{\hat{\text{η}}}_{i,t}+log\underset{c^{\prime }}{\sum^{\text{​}}}e^{{\hat{\text{η}}}_{i,t,c^{\prime }}}\right)\right)$$

As previously mentioned, the voxelwise, classwise, heteroscedastic standard deviations, $\sigma_{i}^{W}$, are predicted by our network in addition to the standard task logits, $f_{i}^{W}$. To accomplish this, we concatenate an additional branch to our architecture immediately following the final upsampling layer. It follows the same architectural structure as the original segmentation branch, with the addition of a softplus activation layer after the last convolutional layer to ensure that all predicted standard deviations are positive.

Under this new formulation, the network is encouraged to assign a high uncertainty to harder-to-segment regions and likewise inclined to assign a low uncertainty to those regions it finds easier to classify.

By modelling heteroscedastic uncertainty in this fashion, we can generate multiple segmentations per input, as for every forward pass through the network, we obtain a new sample from the noise distribution, which is summed with the task logits to produce a new output, where differences are presumed to arise only due to the heteroscedastic uncertainty. As with the epistemic uncertainty quantification, we focus on volumetric differences across the heteroscedastic samples.

#### 3.7.3 Uncertainty quantification

**Fig. 3:**
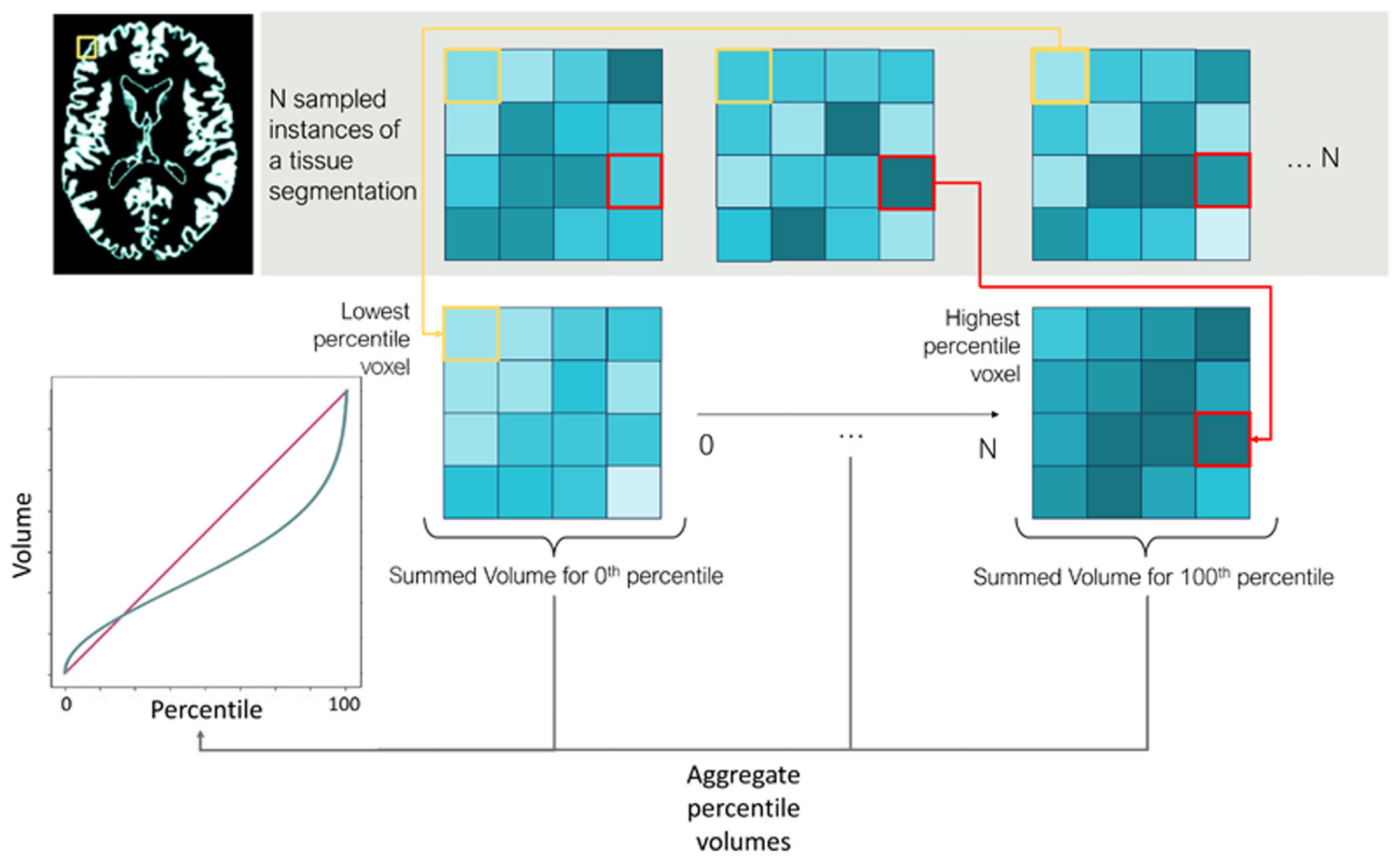
Uncertainty quantification illustrated. The green line in the graph represents the cumulative aggregated percentile volumes, while the red line depicts the cumulative uniform distribution that we aim to fit the former to. This process is repeated for every subject.

To translate sampled segmentations obtained from epistemic and/ or aleatoric modelling into informative quantitative errors, we follow the steps outlined in Eaton-Rosen et al. (2018). For an input image, *x*_*i*_, we perform *T* forward passes through our model with dropout enabled, resulting in *T* predictions per voxel *v*, per class *n*, which we denote as *y*_*itvn*_ for *n* ∈ [1, …, *N*], *v* ∈ [1, …, *V*], and *t* ∈ [1, …, *T*]. We then sort each individual voxel across these *T* Monte Carlo samples according to percentile value, *p*, for *p* ∈ [0, …, 100/*T*], essentially constructing *T* sets of predictions sorted according to voxelwise percentiles, which we denote as λ_*p*_. We then sum the values across all the voxels, which results in a percentile volume, $V_{p}={\text{Σ}}_{v=1}^{V}\lambda_{vp}$, for each percentile prediction. From this, we can construct a cumulative distribution of percentile volumes, which we seek to calibrate. This final calibration step is performed by fitting this cumulative distribution to the cumulative distribution of a uniform distribution. This aims to ensure that confidence intervals reliably contain the expected ratio of ground truth values. The parameters of this fit are calculated on the validation set alone, not to introduce any biases at test time. This quantification process is showcased in Fig. 3.

## 4 Experiments and Results

### 4.1 Section summary

We preface experiment descriptions and results in *Data and experimental details* by describing our data, our initial simulation setup, and outlining how we choose to present our findings. This is followed in *Annealing study: Robustness and quality analysis* by an outline of our first major experiment, namely an ablation study of the methodological additions described in the previous section and resulting findings. *Uncertainty measures and volumetric bounds* outlines the first set of uncertainty-related experiments. Subsection *Physics-driven multi-site harmonisation* contains our primary set of experiments and results, those relating to harmonisation. We first delineate the networks trained and baseline methods, followed by preliminary analyses that investigate basic segmentation quality and biological covariance preservation. We subsequently discuss the main harmonisation-related findings and conduct a scanner-type focused performance analysis.

### 4.2 Data and experimental details

#### 4.2.1 Data

Our data consists of quantitative multi-parametric volumes from 27 subjects originating from a young onset Alzheimer disease dataset (YOAD) (Foulkes et al. (2016), Slattery (2019)). Each volume is 4-dimensional, 1mm isotropic, matrix size 181 × 217 × 181 × 4. The quantitative MR parameters span the fourth dimension. These parameters consist of *R*_1_, the longitudinal magnetisation relaxation rate, $R_{2}^{*}$, the effective transverse magnetisation relaxation rate, proton density (PD), and magnetisation transfer (MT). MT does not feature in the static equation models we employ, so we do not make use of the MT maps. MPM creation details are described in (Helms et al., 2008). For all experiments, we define a train/ validation/ test split of 18/ 4/ 5 subjects, trained over five folds.

#### 4.2.2 Network hyperparameters and training details

Regarding the U-Net component of the network, following the first convolution, we have 30 feature maps at the highest resolution, which are doubled following every contracting block, arriving at 240 at the bottleneck layer. In the decoding branch, the number of feature maps is halved following every expansive block, arriving at 30 immediately prior to the output layer, which reduces this number to *C*, where *C* corresponds to the number of classes. The physics branch takes in a vector of size four (for MPRAGE, constituting TR, TI, *e*^−*TR*^, *e*^−*TI*^) or six (for SPGR, constituting TR, TE, FA, *e*^−*TR*^, *e*^−*T* *I*^, *sin*(*FA*)) parameters and is made up of two fully connected layers of length 40.

Networks are trained with a batch size of four, consisting of 3D patches, each of size 128^3^, uniformly sampled from the synthetically generated images. Networks are trained until convergence, which is met when seven epochs with no improvement in the validation metric have passed. We define the validation metric as the sum of Dice score and volumetric coefficient of variation (calculated per tissue, across images in the batch), averaged across the three tissues.

#### 4.2.3 Simulation sequence details

We seek to evaluate how our additions compare with the original work, and therefore we train our networks using simulated images bearing the same parameter ranges and sequences explored therein. For MPRAGE, this entails TI = [600-1200] ms; for SPGR, this entails TR = [15-100] ms, TE = [4-10] ms, FA = [15-75] degrees. *Physics Gold Standard* labels are generated using the same approach, with the same parameters chosen for the Gaussian mixture models of each tissue.

#### 4.2.4 Results presentation

When comparing models and solutions, statistical significance is ascertained via signed-rank Wilcoxon tests carried out independently on the different metrics. Values in **bold** denote the statistically best models. In instances where models may outperform baselines but are not statistically significantly different from each other, we bold both.

### 4.3 Annealing study: Robustness and quality analysis

We frame this comparative work as an annealing study, whereby we evaluate how model performance changes with each subsequent contribution, verifying their contributions to the model’s efficacy. To this end, we train a series of networks: *Baseline*, a vanilla nn-UNet that takes as input pre-generated data (as in the original work); *Phys-Base*, the physics-informed style network as proposed by the original work, also taking as input pre-generated data; *Aug*, a Baseline network with the shift from using pre-simulated images to training with our proposed physics augmentation pipeline; *Strat*, a *Baseline* model with the added stratification loss across the feature maps in the mini-batch; *Phys-Strat*, equivalent to *Phys-Base* with the added stratification component, also trained with pre-generated data; and *Phys-Strat-Aug*, equivalent to *Phys-Strat* but trained with the augmentation component. We highlight here that our use of *physics-informed* denotes models that are specifically fed the sequence parameters in addition to simulated images.

**Fig. 4:**
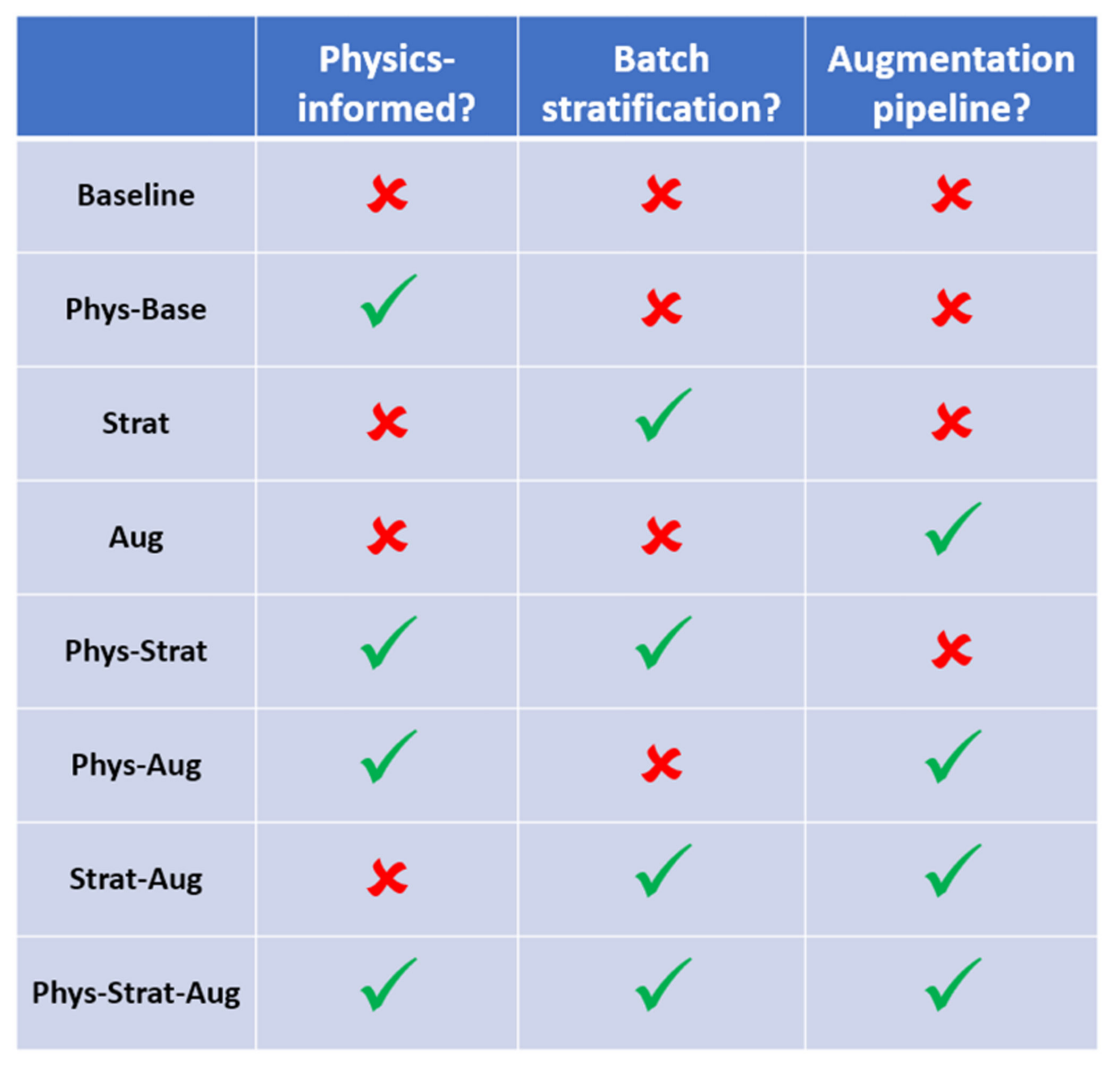
Eight combinations of annealing experiments.

Experiments containing *Phys* are trained with the physics-informed architecture, those containing *Strat* are trained with the added stratification loss across feature maps in the mini-batch, and those containing *Aug* shift from using pre-simulated images to training with our proposed physics augmentation pipeline, taking as input, therefore, 4D MPMs. Combining these terms involves combining these features, and as they operate independently, it does not involve any further modifications. Fig. 4 shows the features that each of the eight experiments contains.

As in the original work, 121 images are simulated per sequence for models trained with pre-generated data. To reiterate, for MPRAGE, equally spaced TI intervals between [600-1200] are chosen for image creation, while for SPGR, the parameters are sampled randomly from the space of TR = [15-100] ms, FA = [15-75] degrees, and TE = [4-10] ms. These parameter ranges are akin to those explored in the original work. Performance is evaluated in terms of volume consistency (using the coefficient of variation, CoV), Dice scores, and Haus-dorff distances. For the *Aug* models, images were simulated using parameters sourced uniformly between the aforementioned ranges.

We posit that a truly physics-informed network should be able to adequately extrapolate to images generated with sequence parameter values that lie outside the range seen during training. To this end, we additionally validate our models on such out-of-distribution (OoD) samples. For MPRAGE, this involves extending the TI range to [100-2000] ms, and for SPGR, TR is expanded to [10-200] ms while TE is expanded to [2-20] ms, and FA is expanded to [5-90] degrees. The same performance metrics are employed to assess performance.

Hausdorff distances, Dice scores and CoV for each experiment and tissue are shown in Table 1, Table 2, and Table 3. CoV results showcase incremental improvements most evidently, with the lowest CoVs belonging in almost all instances to experiments that include the stratification component, which is congruent with expectations, as it is an explicit volume consistency-related constraint. Hausdorff distances paint a similar picture, with the best performances exhibited by *Phys-Strat-Aug* and *Strat-Aug*.

**Fig. 5:**
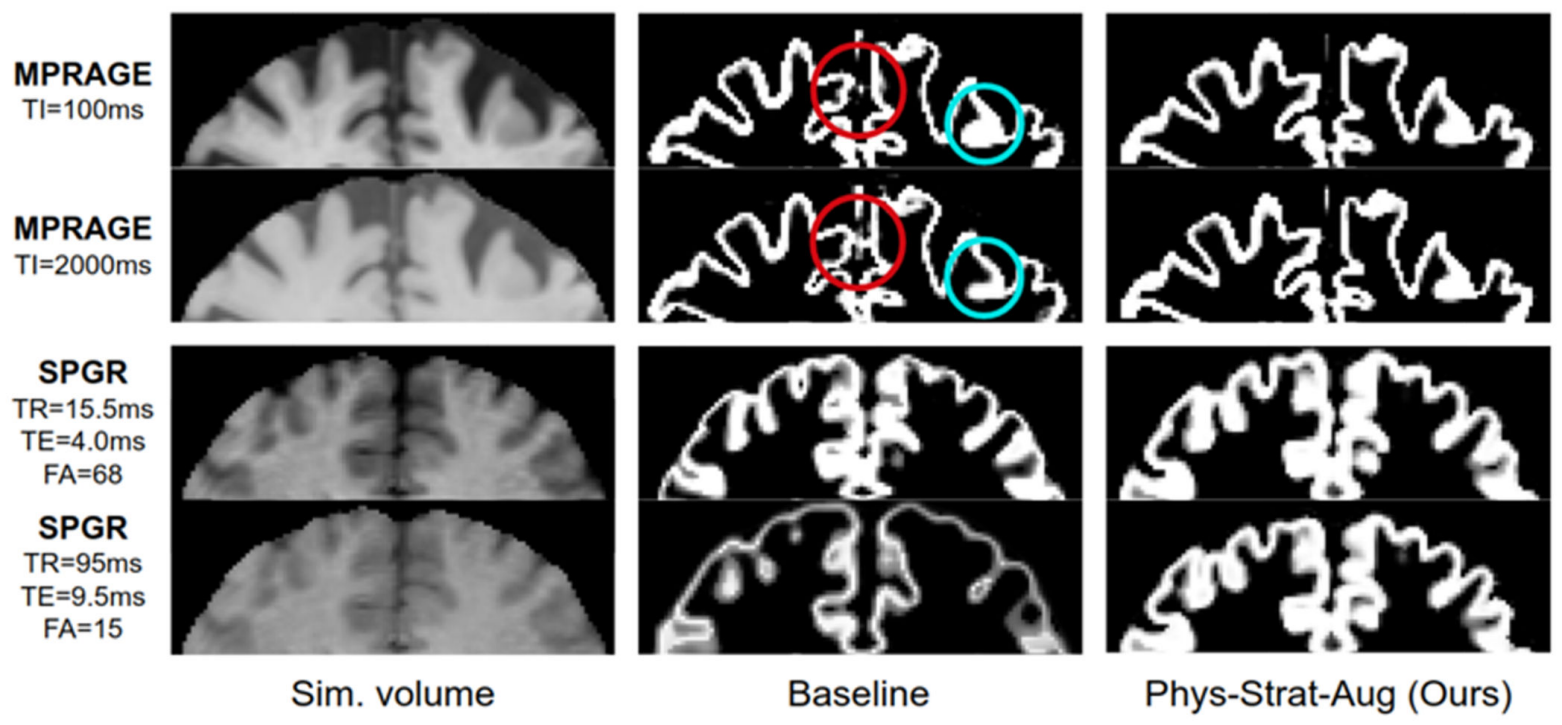
*Baseline* and *Phys-Strat-Aug* comparisons. Comparing out-of-distribution MPRAGE (Top two rows) and SPGR (Bottom two rows) GM segmentations from the proposed and baseline methods. Blue circles highlight examples of significant gyrus variability. Red circles denote regions of segmentation differences between protocols.

We feature comparative qualitative segmentation results in Fig. 5 to showcase how consonant *Phys-Strat-Aug*’s segmentations are across varying contrasts, compared to *Baseline*. We circle specific regions where this is particularly evident.

### 4.4 Uncertainty measures and volumetric bounds

We use the annealing study performances to inform network selection for our uncertainty investigation. *Phys-Strat-Aug’s* performance stands out as the best of the models, so we, therefore, train two sets of uncertainty-aware networks; an epistemic *Phys-Strat-Aug* network, an epistemic *Baseline* network, a heteroscedastic *Phys-Strat-Aug* network, and a heteroscedastic *Baseline* network. The intent is to ascertain how uncertainties deviate for a physics-informed network compared to a physics-agnostic counterpart with respect to in and out of distribution samples.

To extract and calculate volumetric uncertainties we begin by sampling 50 segmentations from our epistemic networks, and 50 segmentations from our heteroscedastic networks, for in and out of distribution samples for MPRAGE and SPGR. Initial comparisons between networks made it apparent that the variance originating from heteroscedastic uncertainty was dwarfed by that originating from its epistemic counterpart by several orders of magnitude. Eaton-Rosen et al. (2018) also made this observation in their work that investigated the translation of uncertainty into quantitative error bounds, finding that the heteroscedastic contribution was negligible. As a result, our quantitative error analyses will henceforth focus solely on epistemic uncertainty.

**Fig. 6:**
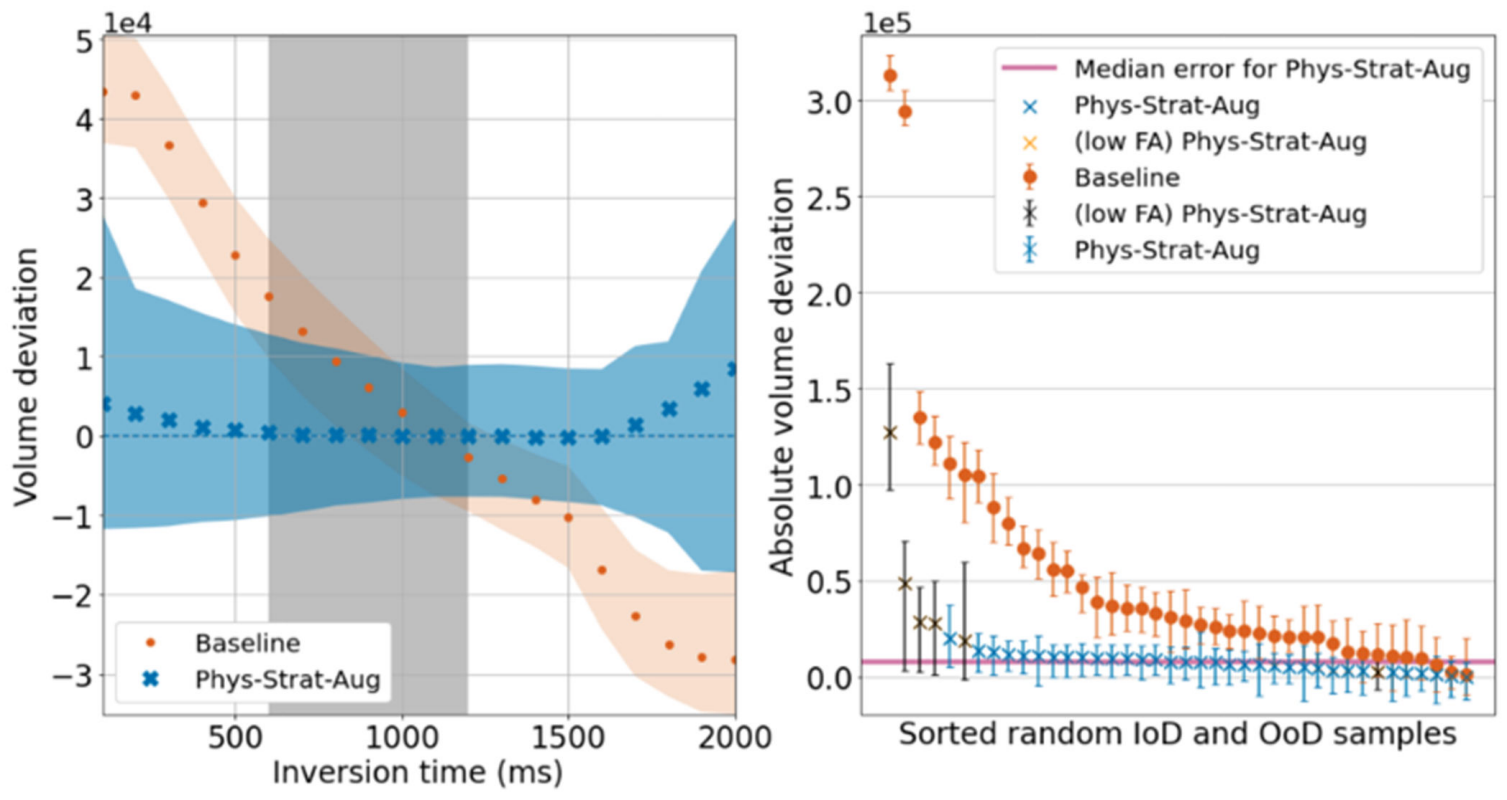
Comparing volume consistency for WM for *Baseline* and *Phys-Strat-Aug*, for an example subject. Filled plots/ Error bars correspond to IQR volumes. Left: MPRAGE. The dashed grey region denotes the TI training time parameter range (600 - 1200 ms), with TIs outside this range being designated as out-of-distribution. Right: SPGR. Shown are the volume deviations from forty realisations of a single subject, where acquisition parameters were sourced randomly from both in and out-of-distribution parameter ranges. Points are sorted according to descending absolute volume deviation. The black points denote samples with FA lower than 10 degrees, for *Phys-Strat-Aug*.

We translate these sampled segmentations into quantitative errors according to subsection 3.7.3. Fig. 6 shows the volume variations for in and out of distribution inference samples, for white matter, for MPRAGE (Left) and SPGR (Right), accompanied by the interquartile range (IQR) volumetric error bounds. For SPGR, the parameter space from which samples are taken is three-dimensional, so for visualisation purposes, we order values according to the absolute volume error. We verify that in both instances, *Phys-Strat-Aug* exhibits significantly more consistent volumes, both in and out of distribution.

Uncertainty-wise, we note that volumetric uncertainties are larger for out-of-distribution samples segmented by *Phys-Strat-Aug* compared to *Baseline*, for both MPRAGE and SPGR, where uncertainties remain more consistent throughout. This is especially evident when looking at the more extreme MPRAGE inversion time samples. It is interesting to note that even for in-distribution images, *Phys-Strat-Aug* exhibits a higher volumetric uncertainty than *Baseline*. We argue that this is because the physics-agnostic *Baseline* is overconfident in its predictions. This network is privy only to images and, as such, can only make predictions based on this singular piece of data. On the other hand, *Phys-Strat-Aug* considers another component, the physics of acquisition, which provides another “axis” along which it can be uncertain. While this grants it a higher uncertainty, we emphasise how the mean segmentation is still high quality and minimises deviation. What this means in terms of utility is that one should look at relative changes in the uncertainty to ascertain in and out of “distributioness” rather than at fixed thresholds.

By focusing on the outliers, we observe that for SPGR samples, uncertainty bounds are noticeably larger for *Phys-Strat-Aug*. These outliers largely correspond to out-of-distribution samples, specifically images simulated with flip angles lower than (10°). As with MPRAGE, the increased error for these samples in *Phys-Strat-Aug* allows most measurements to still overlap with the ground truth volume.

For SPGR, all the apparent outliers for *Phys-Strat-Aug* have significantly larger associated errors, while this is not the case for the *Baseline*. We observe that most outliers correspond to out-of-distribution samples using very low flip angles (< 10°, highlighted in black in the figure). Such images will be significantly less *T*_1_-weighted and therefore be less familiar to the models, in addition to having reduced contrast, resulting in poorer segmentation quality, so the observation that the physics-informed network’s uncertainty around these samples is larger fits with expectations.

### 4.5 Physics-driven multi-site harmonisation

To assess the performance of the method in a multi-site research study setting, we use the ABIDE (Autism Brain Imaging Data Exchange) neuroimaging dataset (Martino et al., 2013), which consists of structural and functional MR images from 19 sites, for 1112 subjects, 539 of which have been diagnosed with Autism Spectrum Disorder (ASD), and 572 of which are controls. We focus on the 11 sites that employed 3D MPRAGE acquisitions, resulting in 614 relevant images. Crucially, the sequence parameters employed across these sites differ, resulting in contrast differences between sites (in addition to other sitespecific effects). The details of the acquisition parameters at each of these 11 sites can be found in Table 4.

By testing our physics-based segmentation methodology on this subset of the ABIDE dataset, our goals are twofold: To further demonstrate that networks trained on synthetic, MPM-based MR data, paired with a robust augmentation scheme, can generalise to data acquired at various different sites; and to show that the standardisation provided by accounting for the physics of acquisition at each site can result in harmonisation that is comparable to or exceeds the performance of existing harmonisation methods.

#### 4.5.1 Reformulating the MPRAGE static equation

The MPRAGE networks discussed thus far have been trained using images generated with only a varying TI. The MPRAGE static equation can also model the effects due to delay time, TD, and echo spacing time, τ, which, when combined with TI, defines TR (Wang et al., 2014):

(10) TR=TI+TD+τ

The MPRAGE static equation can therefore be re-written to incorporate TR directly:

(11) $$b_{M}(x)=G_{M}PD(x)\left(1-\frac{2e^{\frac{-TI}{T_{1}(x)}}}{1+e^{\frac{-TR}{T_{1}(x)}}}\right),$$

This allows us to directly incorporate the two main varying MPRAGE ABIDE site sequence parameters, TI and TR, into the augmentation simulation pipeline, which the network should learn to become robust to. Because TR is contingent on TI, we opt to model a pseudo parameter which we denote pTD, the sum of TD and τ, which is added to TI to create TR.

#### 4.5.2 Main proposed models

To this end, we train three networks: A *Phys-Strat-Aug* style network trained with MPRAGE images generated with TI = [600-1200] ms, and pTD = [500-1600] ms (which leads to TR = [1100-2800] ms), *Strat-Aug*, a network trained akin to *Phys-Strat-Aug*, without the explicit passing of acquisition parameters during training, and *CNN Baseline*, an *Aug* style network trained with MPRAGE images generated with a single set of sequence parameters, to mimic the training of a standard CNN with images originating only from a single site. Additionally, we adopt the same robust augmentation scheme as outlined in Billot et al. (2021) to improve generalisability for all three networks. Namely, these are affine transforms, free-form deformations, spatial blurring, and bias field. We omit the gamma intensity augmentation because it directly affects contrast, which is undesirable in an environment where we want contrast to be strongly associated with imaging parameters. Crucially, we do not employ skull-stripped MPMs as input for training these networks, as we want to show that our models can generalise to un-skull-stripped acquisitions.

#### 4.5.3 Comparison methods

We used SPM12 (r7771, www.fil.ion.ucl.ac.uk/spm/), a widespread neuroimaging processing tool, running on MATLAB (R2019a, The MathWorks Inc., Natick, MA), to resample and rigidly co-register the images into a common space prior to any training. We also employ *SPM12* to generate grey matter, white matter, and CSF segmentations of these images, acting as our non-CNN baseline.

As an additional baseline we select Billot et. al.’s *SynthSeg. SynthSeg* was shown to generalise to unseen data without requiring further training samples from said dataset, regardless of contrast. The authors have made their code and pretrained models readily available for use under a GitHub repository (https://github.com/BBillot/SynthSeg), which we make use of. *SynthSeg* outputs 31 individual parcellations, so to ensure compatibility with our coarser tissue analyses we combine the smaller regions to make up grey matter (cerebral cortex, cerebellum cortex, thalamus, caudate, putamen, hippocampus, amygdala accumbens), white matter (cerebral white matter, cerebellum white matter, pallidum, ventral DC, brain stem), and CSF (lateral ventricles, inferior lateral ventricles, third ventricle, fourth ventricle). Since this implementation of *SynthSeg* does not extra-ventricular CSF, we take intra-cranial masks derived using GIF (Cardoso et al., 2015) (So as not to bias it towards any method used in the study) and make the reasonable assumption that the non-overlapping regions between the total *SynthSeg* parcellations and this mask correspond to the extraventricular CSF.

We implement *PSACNN* as a further baseline. This involves fitting GMMs to the three major tissue classes in all the selected ABIDE images and calculating pseudo-parameters that are used for MPM static equation simulations that are used to generate images for training. We use the same MPMs we use for our proposed method for PSACNN, and all PSACNN networks are trained until convergence.

While both *SynthSeg* and *PSACNN* are not designed for implicit harmonisation, we include them in our harmonisation analysis to ascertain how prominent, frequently used methods perform by default. We have seen how physics-absent models like *Strat-Aug* exhibit non-insignificant harmonisation abilities compared to a baseline, so it stands to reason that, similarly, these methods, too, could demonstrate natural harmonisation capabilities even if they are not directly cognisant of the physics of acquisition as our physics-informed networks are.

We compare harmonisation performance with that offered by *ComBat* (Johnson et al., 2007). ComBat models features as a combination of biological covariates (e.g., Age, gender, pathology) and site effects, the latter of which can be subdivided into additive and multiplicative components. This allows ComBat to regress out site effects while preserving biological variability. ComBat has been shown to consistently outperform other harmonisation methods in various applications, including multisite cortical thickness measurement harmonisation (Fortin et al., 2018), and multi-site diffusion tensor imaging data harmonisation (Fortin et al., 2017) and so we consider it to be the current gold standard. In the context of image segmentation, let *y*_*i* *jv*_ denote the un-harmonised prediction for a specific tissue (grey matter, white matter, or CSF in our case) feature (voxels, volumes, etc.) *v*, for site *i*, and subject *j*. ComBat models this value according to the following:

(12) $$y_{ijv}=\alpha_{v}+X_{ij}\beta_{v}+\gamma_{iv}+\delta_{iv}\epsilon_{ijv}$$

where α_*v*_ represents the average value of that feature across all sites and subjects, *X*_*i*_ *j* represents the design matrix for biological covariates, *β_v_* the design matrix’s corresponding featurewise coefficients, γ_*iv*_ denotes featurewise additive site effects, δ_*iv*_ denotes the multiplicative featurewise site effects, and ϵ_*i* *jv*_ are the model residuals.

The harmonised feature values are derived via the following:

(13) $$y_{ijv}^{Harm\;}=\frac{y_{ijv}-\alpha_{v}-X_{ij}\beta_{v}-\gamma_{iv}}{\delta_{iv}}+\alpha_{v}+X_{ij}\beta_{v}$$

While in certain applications, the features employed can be individual voxels, this is unsuitable for our use case. The choice of feature would have to be congruous across all subjects, which would only be the case if all images were non-rigidly aligned. Such an alignment process would prove destructive for the purpose of volumetric analysis, however, making it ill-suited here. Furthermore, the computational cost to construct such a model for 1842 (614 subjects, three tissue segmentations each) 1mm isotropic 3D volumes would make this impractical. As such, we use the individual tissue volumes as our features, *v*, in our ComBat models.

#### 4.5.4 Segmentation accuracy analysis

**Fig. 7:**
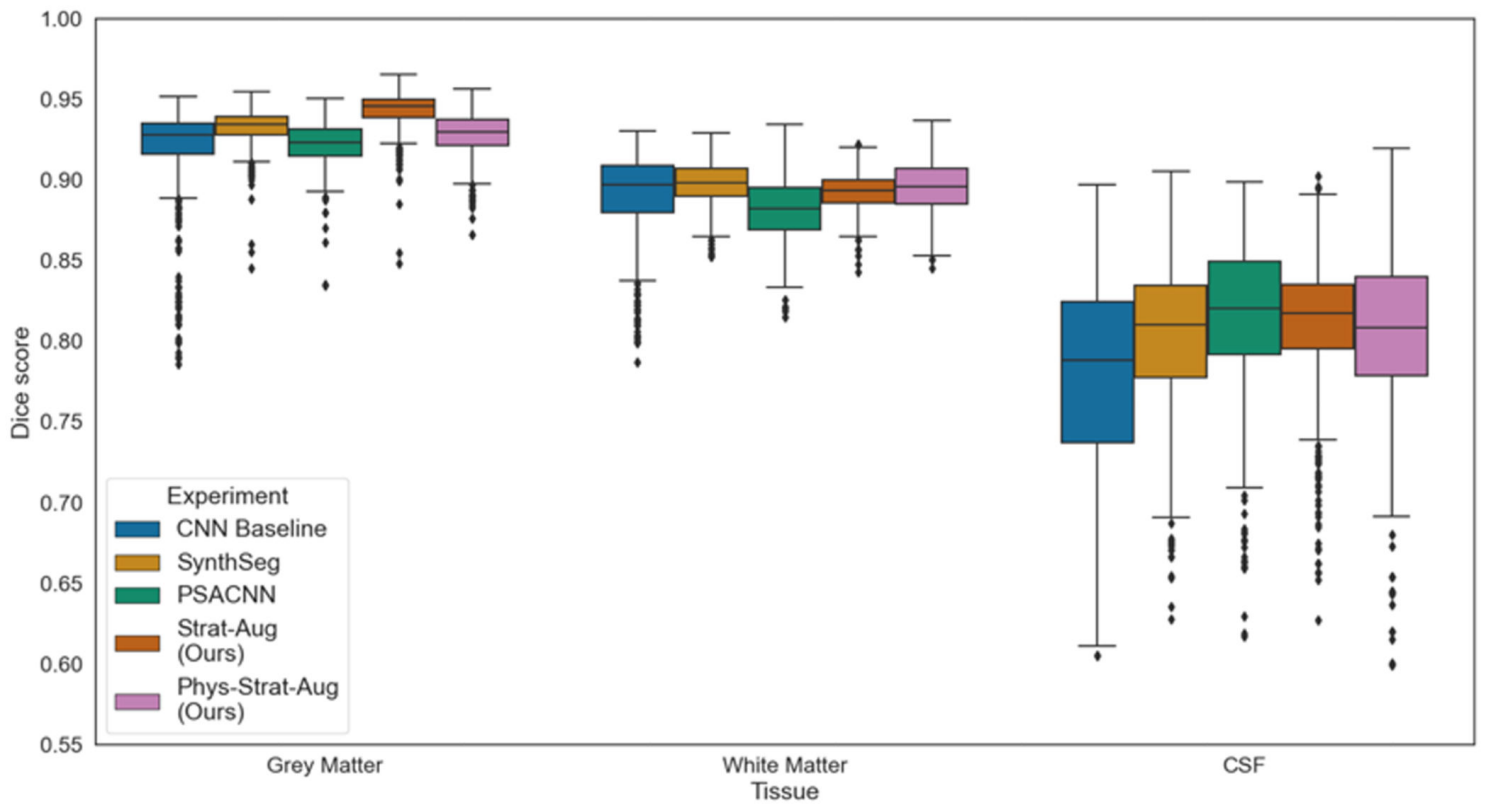
Dice scores of *Phys-Strat-Aug, Strat-Aug, CNN Baseline, PSACNN*, and *SynthSeg* when compared against *SPM*.

As neural networks are often criticised for their unstable behaviour when applied to out-of-distribution data, to ascertain that the proposed method’s segmentation is stable to OoD data, we calculate Dice scores between the segmentation outputs of the proposed models and SPM. SPM has seen widespread use for image segmentation due to its stability, combining mixture models, anatomical spatial priors, and MR-related intensity non-uniformity corrections to classify tissues (Ashburner and Friston, 2005), which justifies its use as a good non-CNN segmentation baseline. Fig. 7 shows the Dice scores calculated between each of the neural network’s predictions and the corresponding SPM segmentation. We do not expect a perfect correspondence, especially when we expect any network exposed to our physics simulation-based methodology to account for the physics of acquisition, but these effects should not influence Dice scores significantly. *CNN Baseline* comparatively exhibits the greatest number of outliers, or failure cases, as well as a greater variance that skews towards lower Dice scores. Dice scores for CSF are notably worse than for grey and white matter. *SynthSeg* exhibits the statistically highest Dice scores for grey matter, *Phys-Strat-Aug* the best Dice scores for white matter, and *PSACNN* the best Dice scores for CSF.

#### 4.5.5 Covariate preservation analysis

Any harmonisation procedure carries with it the risk of masking the effects due to biological covariates (Zhao et al., 2019). A method that removes the effects due to the site but does not ensure that other covariates are preserved is entirely lackluster. ComBat is explicitly designed to attempt to mitigate any deleterious effects its harmonisation procedure might have on covariates, and it follows that we should evaluate our methods to verify that this is also the case. Volume ratios should be predictive of age, so we select age as the variable of interest. We investigate the degree to which age is preserved by fitting a linear age prediction model over the features for a subset of subjects and testing this model on a holdout subset over ten folds. The better the model’s predictive ability, the less the harmonisation process should’ve influenced the age-related biological variability. We hope that our methods preserve age at least as well as ComBat harmonised SPM.

**Fig. 8:**
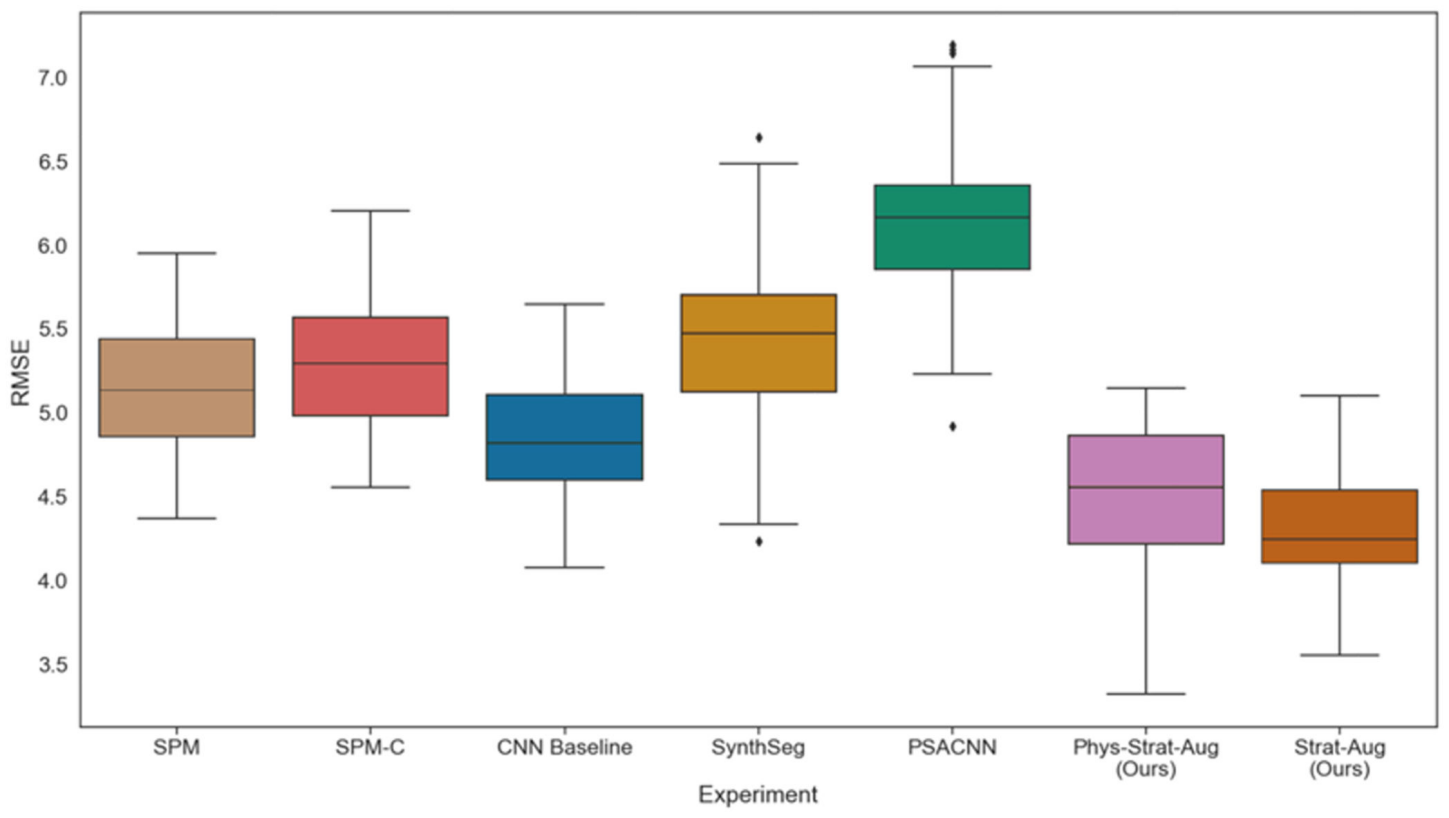
Root-mean-square error boxplots for age prediction using linear regression, for each of the harmonisation experiments. Tissue volumes (GM, WM, and CSF) are used as model features.

Fig. 8 showcases boxplots from the age regression models. *Strat-Aug* boasts the lowest RMSE, while ComBat-harmonised SPM (SPM-C) exhibits the worse performance. It is interesting to note that *Phys-Strat-Aug* performs worse than its physics-agnostic counterpart. While the difference is not large and still outcompetes alternative methods, it could be explained by the fact that the physics-informed model overcompensates when accounting for the sequence variables. To elaborate, physics-informed models adjust their predictions based on the physics parameters fed. The model’s knowledge of the physics parameters is not expected to be perfect; there are additional unmodelled variables, absent in the static equation formulations, that the model is not made privy to (scanner make, site of acquisition, noise profiles, etc.) that affect contrast that the model has to account for implicitly. The erroneous conflation of these non-systematic effects with the effects that arise purely from the physics of acquisition can lead to the model exaggerating the degree to which it tries to correct them, given the information it is exposed to, which could explain these observed discrepancies.

#### 4.5.6 Multi-site harmonisation analysis

The age distribution is not homogeneous across all sites, so it is not reasonable to assume that age-based trends between age-heterogeneous site distributions should be alike. We, therefore, partition the sites into two distinct groups based on a per-site mean age. The first we denote as “Young”, where sites whose mean age is less than 16 are selected. The sites that fulfill this criteria are *OHSU, UCLA*_1_, *UCLA*_2_, *YALE*, and *NYU*. The second partition we denote as “Old”, where sites whose mean age is greater than 22 are selected. The sites that fulfil this criterion are *CALTECH, USM*, and *CMU*. Under these criteria, *PITT* is excluded.

Per model (for each site and tissue), per age-partitioned group, we use linear regression to fit a linear trend. Table 5 shows the mean and standard deviations of these trends across sites, per group, tissue type, and experiment. We expect that if the volumes are well-harmonised, the intercepts and gradients across all sites should be similar. The degree of similarity should be reflected in the standard deviation of these variables across all sites.

We make use of Levene’s test to ascertain whether the standard deviations between each experiment’s trends and those of *SPM-C* are significant. We verify that *Phys-Strat-Aug* and *Strat-Aug* exhibit statistically significantly lower inter-site standard deviations than *SPM-C* for grey matter and CSF, for most instances of slope and intercept. *CNN Baseline*, on the other hand, does not perform better than *SPM-C* in most cases and, in fact, shows noticeably worse intercept standard deviations, in particular for white matter in the “Old” cohort. Additionally, the mean trend gradients predicted from *CNN Baseline’s* segmentations present the greatest departure from its counterparts when looking at the “Young” cohort.

We further investigate the changes incurred by fitting Com-Bat models to the segmentation volume values of each of the CNN-based experiments. We note that fitting a ComBat model on all the CNN-based model outputs results in decreased intercept standard deviations but slightly worse gradient standard deviations. This is unsurprising, as accounting for the additive effects of site (i.e., any linear discrepancies which relate to the linear intercept) is the easier of the tasks to perform. Still, it seems to come at the cost of gradient variation. Unsurprisingly, *CNN Baseline* benefits the most from this process, as its site volumes displayed the greatest disparity in most cases.

**Fig. 9:**
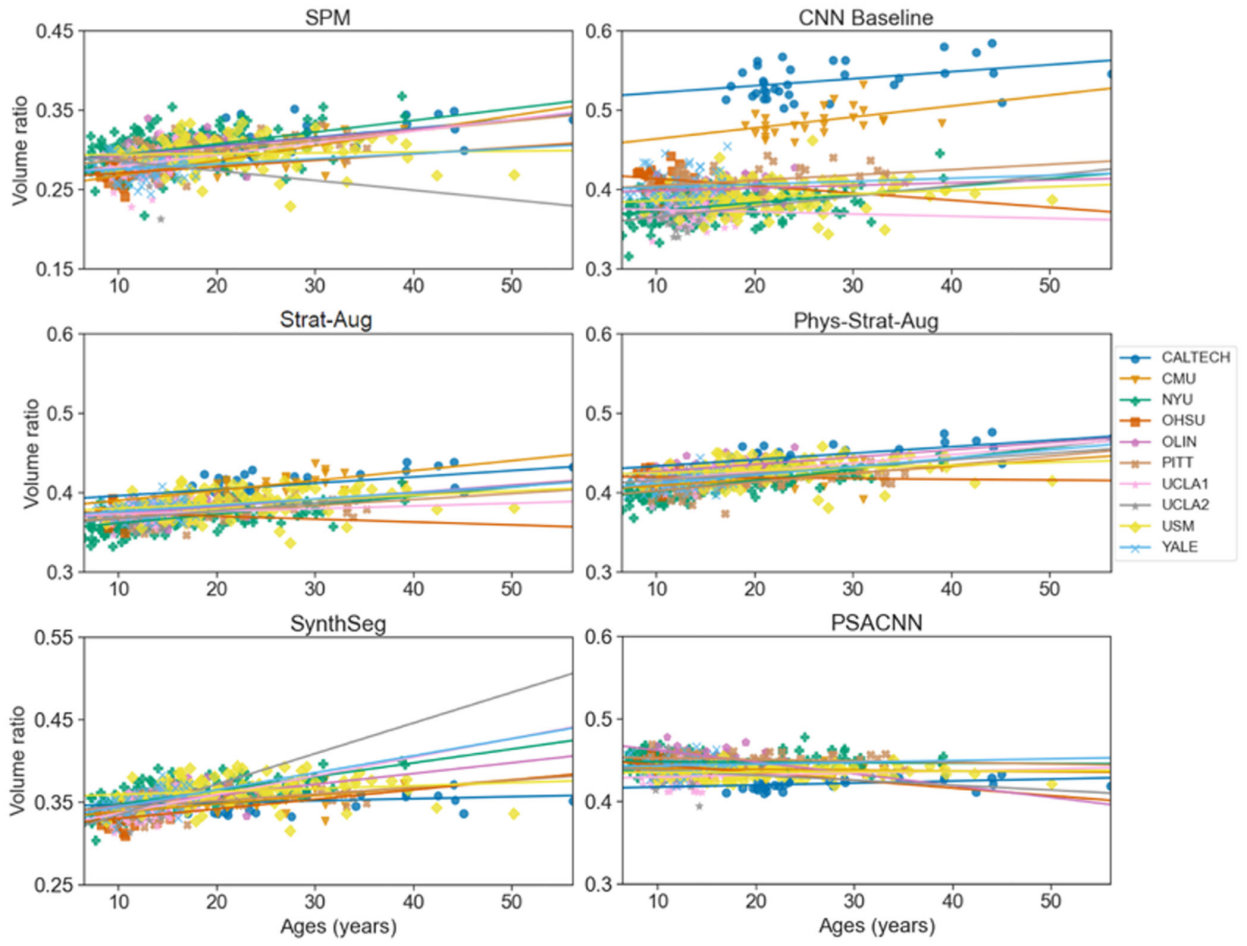
White matter volume ratios for all MPRAGE subjects in the ABIDE dataset for each of the experiments. Linear trends of best fit are calculated and shown, per site, extrapolated to the full dataset age range.

Fig. 9 shows a scatter plot against age of white matter volume ratios for all subjects for all the CNN-based experiments. Qualitatively, this figure clearly shows that *CNN Baseline* has completely failed to generalise to two of the sites, as evidenced by their distinctly higher white matter volume ratios compared to other sites. This is not observed for *Phys-Strat-Aug* or *Strat-Aug*, indicating that the physics augmentation pipeline has allowed these networks to generalise to sites exhibiting different tissue contrasts aptly. This is also reflected in Fig. 7, which illustrates CNN Baseline’s greater number of negative outliers, as well as its statistically significantly poorer performance compared to *Phys-Strat-Aug*, for all tissues. Expectedly, *Synth-Seg* aptly generalises to all sites, evidenced by both figures. *PSACNN* shows similar age trends for all sites in Fig. 9, though exhibiting a flatter gradient compared to other methods. In terms of Dice scores, it under-performs on average compared to other methods in terms of grey and white matter (though exhibiting far less extreme outliers when compared to *CNN Baseline*, but outperforms these when it comes to CSF.

#### 4.5.7 Performance across different scanner types

One obvious pitfall for a method trained using static equations is that it might fail to generalise to images acquired on scanners from vendors that employ different sequence implementations. The quantitative data was acquired on a Siemens Magnetom TrioTim scanner, and the formulation of the MPRAGE equation is based on the Siemens MPRAGE sequence.

To assess this degree of generalisability, or lack thereof, we make use of 1233 *T*_1_-weighted images from the Alzheimer’s Disease NeuroImaging Initiative (ADNI^1^). Of these, 682 were acquired on Siemens scanners, 303 on General Electric scanners, and 248 on Philips scanners. We ran inference on these images with *Phys-Strat-Aug, Strat-Aug, PSACNN* (re-trained using predicted pseudo-parameters from these ADNI subjects) and *SynthSeg*. Statistical significance is evaluated based on signed-rank Wilcoxon tests. For *Phys-Strat-Aug*, lacking equivalent exchanges for the parameters for the non-Siemens scanner sequences, equivalent parameter choices are made.

**Fig. 10:**
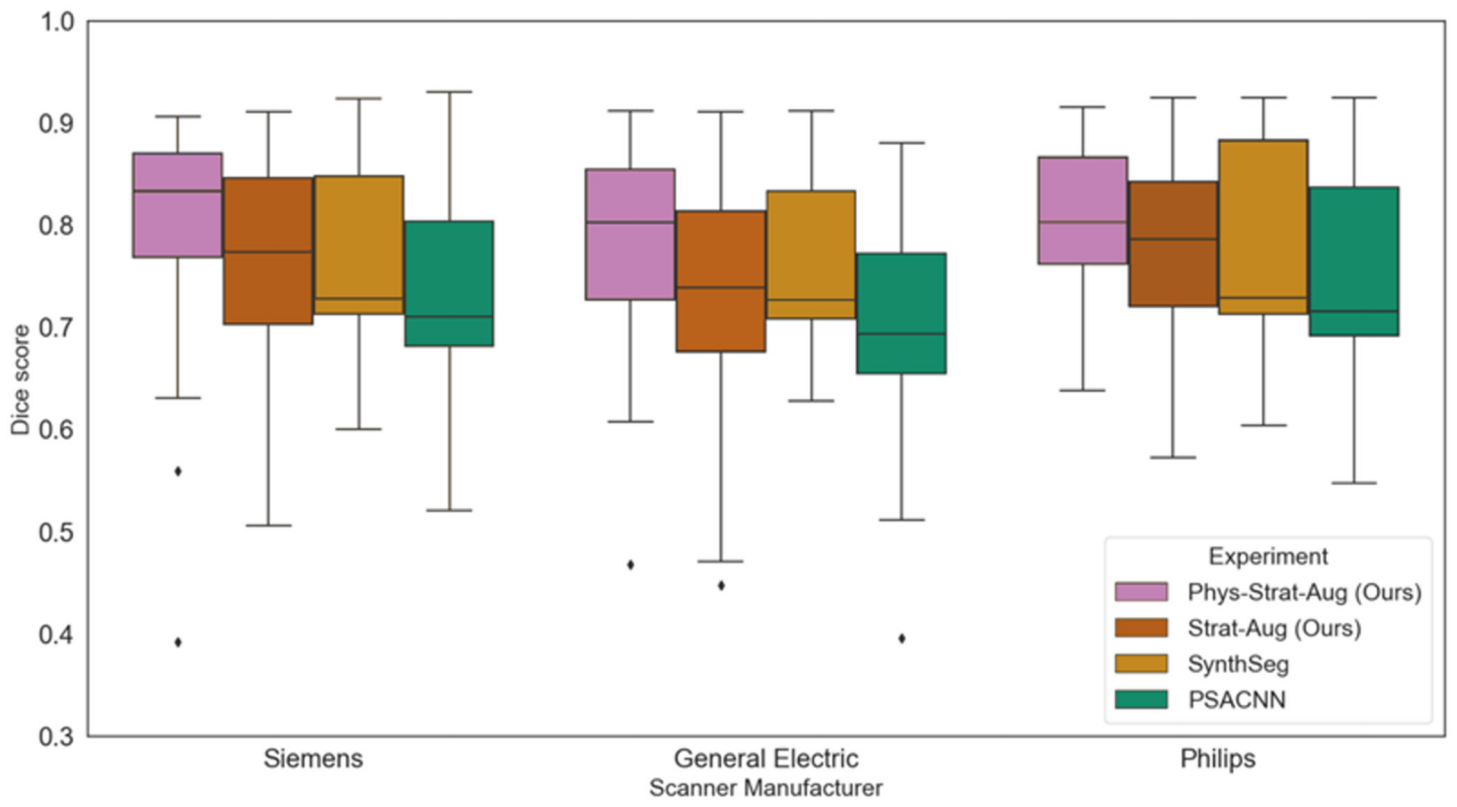
Dice score boxplots for models evaluated on ADNI images originating from three different manufacturers.

Fig. 10 showcases boxplots for the Dice scores for each model and manufacturer. *Phys-Strat-Aug* exhibits the statistically best performance across all scanner types. *Strat-Aug* outperforms *SynthSeg* on those images acquired by Siemens and General Electric scanners, and *PSACNN* performs worst across all scanners.

We verify that for *Phys-Strat-Aug* and *Strat-Aug*, there are statistically significant differences between the Dice scores across all manufacturers, with *Phys-Strat-Aug* performing best on Siemens images, and *Strat-Aug* performing best on Philips images. For *SynthSeg*, this is only observed between Philips and General Electric, but there is no significant difference between Siemens and General Electric or between Siemens and Philips. Quality-wise, *Phys-Strat-Aug* exhibits the statistically highest Dice scores at all sites.

### 4.6 Uncertainty-informed sequence optimisation

In most neuroimaging research studies, MRI images are obtained to extract surrogate biomarkers of interest. These images are thus optimised to maximise signal and contrast (and subsequently good measurements) in the region of interest. As uncertainty is not only proportional to the degree of unfamiliarity but also to the difficulty of the task, an image which results in the lowest segmentation uncertainty of the region of interest should be optimal from the point of view of the surrogate biomarker of interest.

We can therefore leverage uncertainty predictions to ascertain parameter choices or regions in parameter space that minimise said uncertainty. In theory, images produced with these parameter pairs will be easiest for networks to segment and should produce the most confident predictions. Furthermore, we can verify if those regions of minimal uncertainty overlap with typical sequence parameters sourced from real-life studies. To be clear, we don’t carry out this investigation to promote our models as a replacement for standard sequence optimisation practices but more as a validation that our models’ uncertainty matches expectations established by observation.

To this end, we run inference on two uncertainty-aware networks for each sequence of interest, SPGR and MPRAGE, on simulated volumes spanning the range of sequence parameters to be investigated. We then calculate the calibrated volumetric uncertainty for each volume as outlined in previous sections and aggregate the results in a contour plot. The range of parameters spanned for SPGR are (TR) = [5-100] ms and flip angle (FA) = [5-90] degrees. Because we only deal with *T*_1_-weighted images, whose contrast is largely dependent on TR, TE is ideally minimised. As such, it is fixed at 4 ms; furthermore, its inclusion would increase the processing time multiplicatively. Akin to the MPRAGE networks trained for the ABIDE harmonisation, we vary both pTD and TI. The range of parameters spanned for MPRAGE are (pTD) = [200-2000], (TI) = [400-2000].

**Fig. 11:**
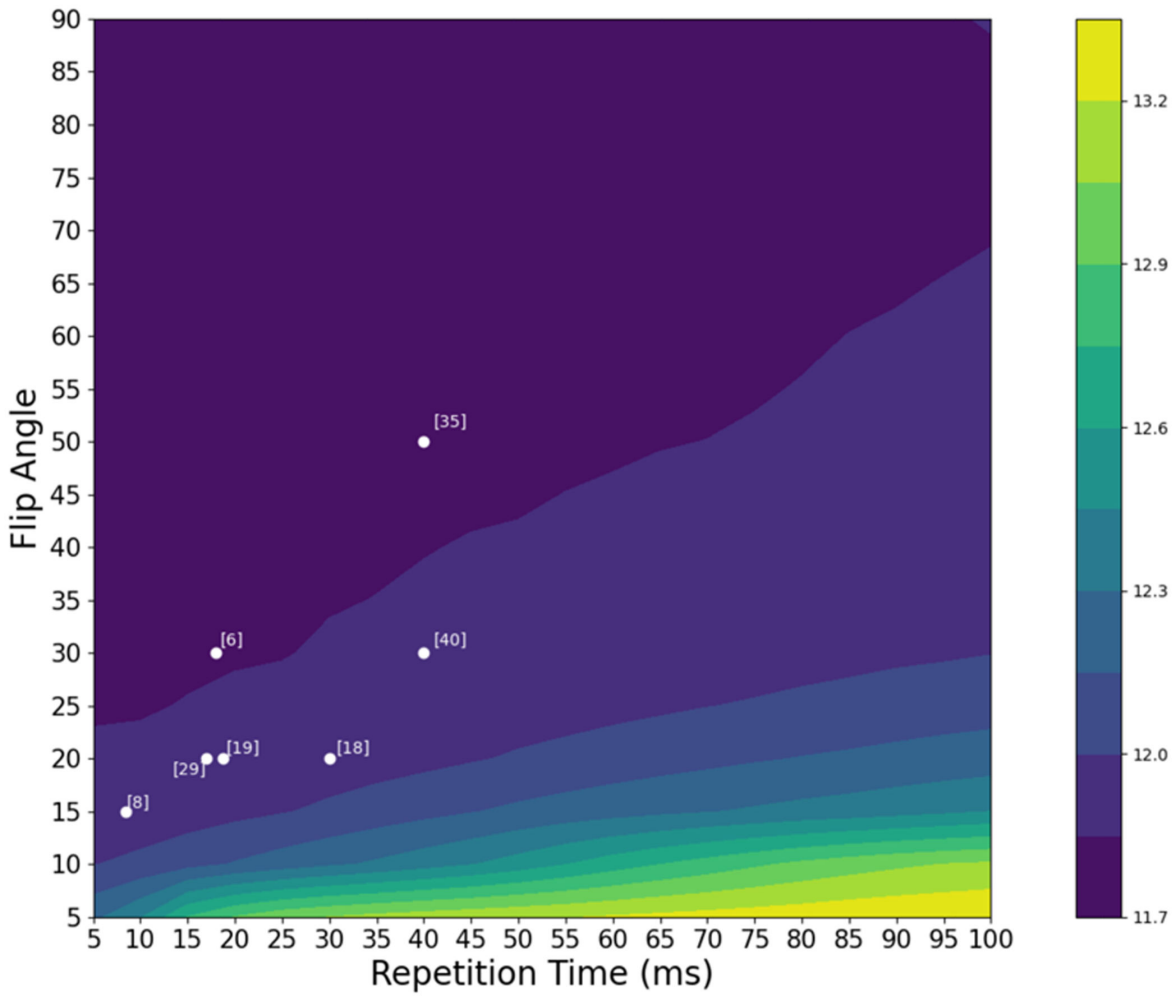
Logarithmic SPGR uncertainty contour, averaged over all tissues and subjects. Scattered white points denote parameter choices sourced from relevant neuroimaging literature. Numbers enclosed in square brackets adjacent to each point denote the relevant reference: [6]: Cocosco et al. (1997), [8]: Di Martino et al. (2014), [18]: Helms et al. (2008), [19]: Helms et al. (2009), [29]: Leow et al. (2007), [35]: Runge et al. (1991), [40]: Taki et al. (2011)

**Fig. 12:**
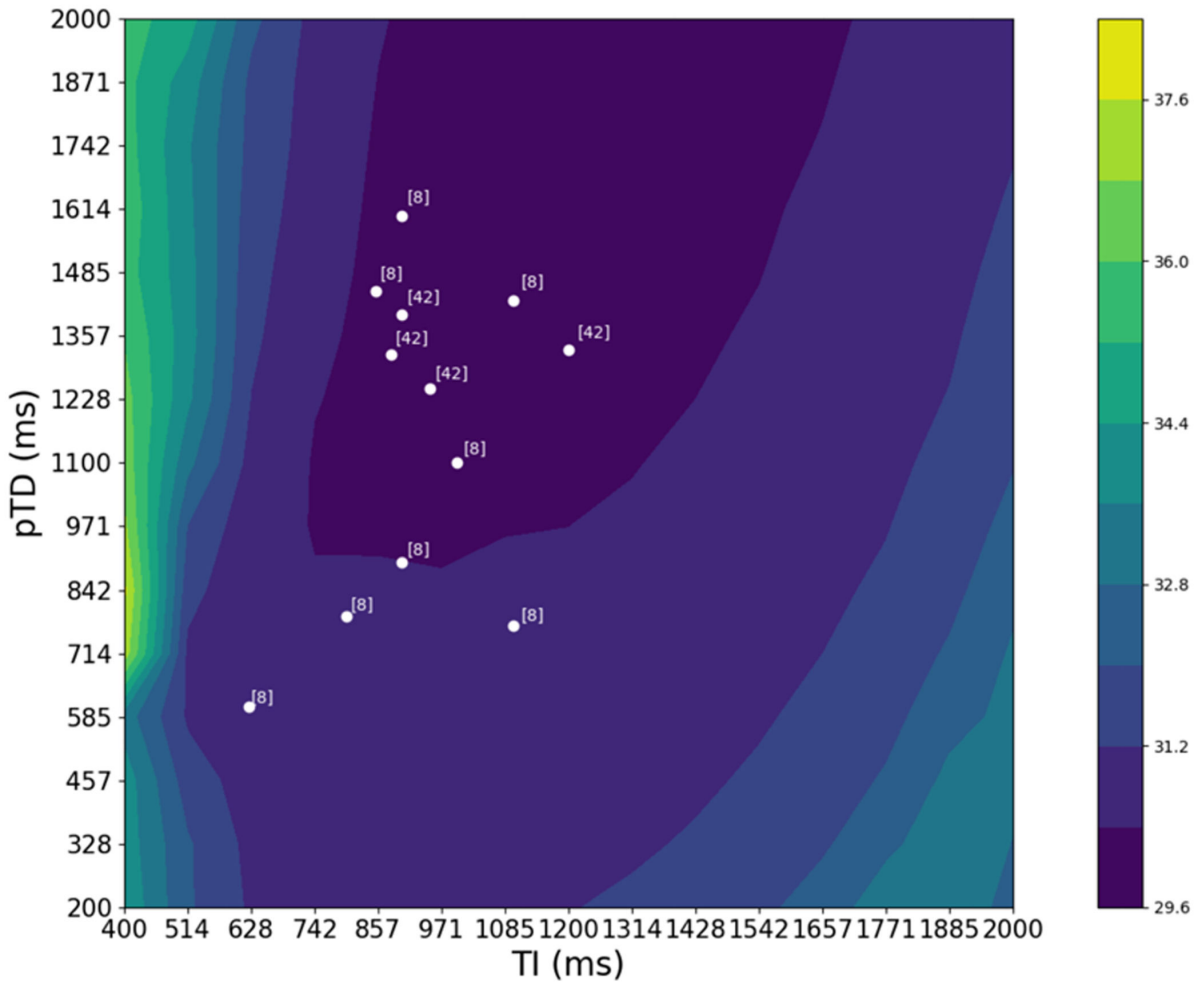
Logarithmic MPRAGE uncertainty contour, averaged over all tissues and subjects. Scattered points denote parameter choices sourced from relevant neuroimaging literature. Numbers enclosed in square brackets adjacent to each point denote the relevant reference: [8]: Di Martino et al. (2014), [42]: Wang et al. (2014)

Fig. 11 and Fig. 12 show the logarithmic volumetric uncertainty contours for SPGR and MPRAGE, respectively, averaged across all tissues and inference subjects. Though not shown, there is a significant overlap between subjects, which lends credence to the notion that the uncertainty is driven by the contrast between tissues, and should therefore be independent of subject-specific anatomy. The scattered points denote the sequence parameters used by various studies and trials, where the annotations correspond to relevant references to these. For MPRAGE, there is a significant overlap between literature sequence parameters and regions of lowest uncertainty.

For SPGR, this is also verified, though there is an absence in the literature of *T*_1_-weighted SPGR sequences employing larger (> 50°) flip angles and larger (> 60 ms) repetition times. Higher repetition times are correlated with decreased *T*_1_-weighting, and increased proton density weighting (Gras et al., 2013). Additionally, if a good contrast can be attained at a lower *TR* then it stands to reason that it would be selected since this would also save time by reducing the total acquisition time (which is proportional to *TR*).

The absence of higher flip angles in *T*_1_-weighted SPGR studies can be explained in the context of Ernst angles, the flip angle at which the signal for a particular tissue is maximised given a certain TR and the *T*_1_ properties of the tissue of interest. For the *TRs* typically employed in *T*_1_-weighted SPGR sequences, the Ernst angle for GM and WM lies in the range of [10-25]° (Runge et al., 1988). Maximising a single tissue’s signal won’t guarantee that the contrast between tissues is maximised, which can result in higher FAs being selected. Far from the Ernst angle for these tissues, the signal falls exponentially, however, even if the relative contrast remains significant, which in turn results in a lower signal-to-noise ratio. Our simulation model does not explicitly account for this, as a noise model is absent, but is well-suited for inclusion in future work.

## 5 Limitations

The method is admittedly limited to those sequences that can be aptly represented as static equations. We do argue that at the very least, for the purposes of contrast agnosticism, a wide enough range of realistic contrasts can be generated with currently implemented sequences, which should allow for our method to generalise further. Furthermore, our method relies on training with quantitative data, which is far more scarce than its qualitative counterpart. This can hinder widespread adoption compared to typical, qualitative image-based CNN pipelines.

The *Physics Gold Standard*, while proving apt for coarse segmentations, is not suitable for obtaining finer parcellations, as it becomes more complicated or even impossible to differentiate between smaller structures using their intrinsic MR properties alone. That being said, there is nothing in the method that precludes the use of standard, finer, manual annotations if the task calls for it, and more consistent parcellations would still be expected, granted that these would no longer be based on anatomical ground truth.

## 6 Discussion and Conclusions

In this work, we demonstrated that with some well-justified modifications to the training pipeline, a physics-informed network can achieve extremely constrained tissue segmentations across a wide range of contrasts across all tissue types and investigated sequences, thus strengthening its harmonisation capabilities.

*Phys-Strat-Aug* boasts the statistically best performance across all metrics for most experiments. In instances where it is outperformed, this almost always corresponds to *Strat-Aug*. It is evident that the stratification loss with its respective mini-batch handling proves to be especially beneficial from the volume consistency it enforces, becoming even more potent when combined with the simulation augmentation, which affords the model a richer exploration of sequence parameter space which should not only grant it greater invariance to the choice of acquisition parameters but also greater generalisability owing to the functionally infinite training data at its disposal.

Furthermore, we also showed that it can suitably generalise to unseen domains while maintaining volume consistency without compromising segmentation quality and is validated by accurately quantifying the volumetric uncertainty. The uncertainty estimates further suggest that incorporating knowledge of the acquisition scheme grants the model an additional level of safety, as volumetric uncertainties proved to be larger for out-of-distribution parameter-generated images.

From our uncertainty-based sequence validation, we verify that, for MPRAGE, there is a significant overlap between the sequence parameter region of lowest uncertainties assigned by our physics models and those sequence parameters employed in the literature for neuroimaging studies. This is a twofold boon, as it not only lends credence to the model by showcasing this congruence but also could also allow for exploratory work involving newly developed sequences. Given their static equation analogue, they could be used in combination with our uncertainty-aware networks to ascertain in advance those parameter combinations that might be most favourable for analyses, in a precursor-style type of analysis rather than a replacement for existing optimisation techniques, of course. The SPGR counterpart showcased that while the minimum uncertainty overlap is verified, there are clearly unaccounted-for variables that need to be considered, such as noise modelling.

Our experiments on the multi-site ABIDE dataset showcase how our pipeline can provide harmonised segmentations for a multi-site volume study that largely out-competes the ComBat harmonised SPM baseline while also proving equal to or better at this than another contrast-agnostic method, *SynthSeg*. It is worth noting that *SynthSeg*’s mean normalised volume gradients for grey matter were overall noticeably lower compared to other experiments, and for the “old” cohort, fell outside the range of expected as found in literature trends(Scahill et al. (2003), (Farokhian et al., 2017)), potentially compromising its ability to be used in such a fashion. The same is observed for *PSACNN*, in an even more extreme manner for grey and white matter.

Furthermore, we demonstrate how a naively trained, single-contrast baseline fails to generalise to multiple sites, providing less congruent trends, worse tissue volume ratio matching, and worse segmentation quality. When combined with the observation that the performance between *Phys-Strat-Aug* and *Strat-Aug* is comparable, there exists the implication that the most relevant component in the pipeline is the multi-contrast physics augmentation block. However, small further improvements seem to be achieved by explicitly incorporating the sequence acquisition parameters.

We further emphasise how our physics-informed networks were able to generalise well to a complete holdout dataset composed of images originating from multiple different sites, acquired using different sequence parameters. This is in spite of the low number (27) of unique phenotypes involved in training and validating our models. We attribute this both to our physics augmentation pipeline and to the robust augmentation scheme employed, particularly the use of free-form deformations. However, as mentioned previously, the CSF Dice scores are significantly lower than their GM and WM counterparts. This can, in part, be attributed to how SPM’s CSF maps include non-CSF tissue, such as dura mater, which are absent from the CNN-based experiments’ CSF segmentations, and this drop in performance is also observed for *SynthSeg* and *PSACNN*.

The ABIDE portion of this study evaluates our model in terms of sequence parameter variations, but we also sought to investigate performance across scanners made by different ven-dors. By testing our model on images acquired on Siemens, General Electric, and Philips scanners, we observed how *Phys-Strat-Aug* exhibited high Dice scores when compared to established baselines, *SynthSeg* and *PSACNN*, outperforming both in all instances.

In summary, we demonstrate that physics-informed networks, trained with MR static equation-based simulated data, can generalise to unseen datasets while also alleviating the biases introduced by different sequence parameter choices to produce more consistent segmentations and, therefore, volumetric biomarkers without requiring manual tissue annotations. We also show that these networks, when paired with uncertainty modelling, are capable of use as sequence parameter investigation models.

Future work will involve using finer parcellations, not directly derived from the quantitative maps themselves in the same fashion as the *Physics Gold Standard*, and investigating the degree to which segmentation consistency can be attained in these instances. Furthermore, we would like to explore the effects of varying input image resolution, as this is a property that data sourced from the clinic often boasts.

## Supplementary Material

Appendix A

## Figures and Tables

**Table 1:** Mean directed Hausdorff distances for *Baseline, Phys-Base, Aug, Strat, Phys-Strat, Phys-Aug, Strat-Aug*, and *Phys-Strat-Aug* on segmentation task, across inference subjects. All Hausdorff distances are calculated against a Physics Gold Standard. Standard deviations quoted in brackets. Bold values represent statistically best performances.

	Sequence Hausdorff distances							
Experiments	MPRAGE				SPGR			
	GM		WM		GM		WM	
	IoD	OoD	IoD	OoD	IoD	OoD	IoD	OoD
Baseline	9.02 (1.70)	13.44 (1.58)	10.48 (3.00)	15.36 (3.21)	8.30 (0.65)	22.43 (7.56)	7.81 (0.28)	27.79 (4.98)
Phys-Base	4.86 (0.90)	5.45 (0.82)	5.95 (0.86)	6.19 (0.70)	8.73 (1.46)	34.03 (7.71)	8.16 (0.59)	31.35 (7.33)
Aug	6.46 (2.72)	6.65 (2.59)	6.08 (0.45)	6.19 (0.45)	7.02 (2.58)	37.39 (19.95)	19.08 (7.15)	43.64 (46.25)
Strat	6.13 (0.92)	6.24 (0.90)	6.95 (0.003)	6.96 (0.69)	**4.99** **(2.48)**	7.21 (2.79)	5.81 (1.21)	8.02 (3.48)
Phys-Strat	5.67 (1.54)	5.66 (1.44)	5.40 (0.77)	5.39 (0.78)	6.02 (1.43)	6.77 (3.04)	6.09 (0.90)	7.53 (4.39)
Phys-Aug	5.74 (1.39)	6.13 (1.56)	6.74 (1.20)	6.85 (1.13)	7.32 (2.63)	11.08 (9.03)	8.67 (1.64)	34.99 (14.01)
Strat-Aug	5.85 (3.02)	5.96 (2.98)	4.71 (0.93)	4.79 (0.76)	5.27 (1.02)	**4.19** **(0.77)**	**4.55** **(0.92)**	**4.66** **(0.87)**
Phys-Strat-Aug	**3.17** **(0.23)**	**3.20** **(0.21)**	**3.52** **(0.81)**	**3.59** **(0.72)**	**4.98** **(1.08)**	6.16 (0.89)	6.00 (0.55)	6.13 (0.45)

**Table 2:** Mean Dice scores for *Baseline, Phys-Base, Aug, Strat, Phys-Strat, Phys-Aug, Strat-Aug*, and *Phys-Strat-Aug* on segmentation task, across inference subjects. All Dice scores are calculated against a Physics Gold Standard. Standard deviations quoted in brackets. Bold values represent statistically best performances.

	Sequence Dice Scores							
Experiments	MPRAGE				SPGR			
	GM		WM		GM		WM	
	IoD	OoD	IoD	OoD	IoD	OoD	IoD	OoD
Baseline	0.966 (0.005)	0.956 (0.006)	0.953 (0.002)	0.934 (0.002)	0.878 (0.021)	0.872 (0.008)	0.893 (0.023)	0.873 (0.011)
Phys-Base	**0.971** **(0.007)**	0.964 (0.009)	**0.964** **(0.008)**	**0.959** **(0.011)**	0.911 (0.020)	0.872 (0.050)	0.912 (0.021)	0.880 (0.092)
Aug	0.967 (0.004)	0.964 (0.004)	0.960 (0.002)	0.958 (0.002)	0.916 (0.019)	0.892 (0.025)	0.900 (0.004)	0.874 (0.035)
Strat	0.958 (0.005)	0.957 (0.005)	0.946 (0.003)	0.945 (0.003)	0.928 (0.002)	0.901 (0.010)	0.911 (0.005)	0.877 (0.009)
Phys-Strat	**0.970** **(0.005)**	**0.969** **(0.005)**	0.958 (0.004)	**0.957** **(0.005)**	0.929 (0.015)	0.911 (0.011)	0.922 (0.021)	0.894 (0.040)
Phys-Aug	0.968 (0.003)	0.966 (0.003)	0.960 (0.003)	0.958 (0.003)	0.930 (0.002)	0.893 (0.004)	0.919 (0.004)	0.874 (0.003)
Strat-Aug	0.960 (0.004)	0.959 (0.003)	0.951 (0.003)	**0.960** **(0.003)**	**0.936** **(0.006)**	**0.920** **(0.005)**	**0.935** **(0.004)**	**0.897** (0.004)
Phys-Strat-Aug	**0.971** **(0.004)**	**0.971** **(0.005)**	**0.962** **(0.003)**	**0.960** **(0.004)**	0.930 (0.016)	0.913 (0.019)	0.921 (0.015)	**0.899** **(0.019)**

**Table 3:** Coefficients of variation (CoV) for *Baseline, Phys-Base, Aug, Strat, Phys-Strat, Phys-Aug, Strat-Aug*, and *Phys-Strat-Aug* on segmentation task, averaged across test subjects. Standard deviations quoted in brackets. Bold values represent statistically best performances.

	Sequence CoVs (x10^3^)							
Experiments	MPRAGE				SPGR			
	GM		WM		GM		WM	
	IoD	OoD	IoD	OoD	IoD	OoD	IoD	OoD
Baseline	6.39 (0.87)	22.50 (4.08)	14.94 (1.71)	51.12 (7.11)	61.91 (7.61)	170.10 (31.32)	32.57 (11.98)	158.93 (16.83)
Phys-Base	2.72 (2.12)	14.67 (7.30)	3.28 (2.01)	28.10 (3.98)	77.22 (34.44)	127.22 (18.61)	20.77 (9.35)	264.80 (8.52)
Aug	2.81 (1.26)	8.79 (2.42)	2.16 (0.67)	10.27 (3.20)	21.00 (5.91)	266.82 (15.99)	17.76 (9.42)	258.28 (18.71)
Strat	1.27 (0.29)	**4.15** **(1.56)**	1.33 (0.51)	5.05 (2.36)	17.68 (3.01)	29.91 (8.02)	8.88 (2.71)	52.17 (9.19)
Phys-Strat	0.71 (0.23)	6.15 (1.51)	0.53 (0.25)	**3.67** **(1.34)**	21.83 (0.83)	59.78 (13.31)	8.60 (0.64)	59.19 (11.25)
Phys-Aug	2.66 (0.94)	10.73 (0.53)	3.47 (1.33)	7.78 (1.51)	**9.54** **(5.45)**	31.00 (9.02)	6.70 (3.33)	88.62 (18.32)
Strat-Aug	2.13 (0.84)	6.98 (2.67)	**0.35** **(0.14)**	4.28 (1.01)	13.25 (5.58)	35.97 (7.46)	**3.69** **(1.76)**	46.95 (13.55)
Phys-Strat-Aug	**0.42** **(0.22)**	4.74 (1.30)	0.51 (0.23)	**3.65** **(0.62)**	15.76 (1.18)	**28.88** **(9.74)**	7.12 (0.45)	**44.78** **(4.22)**

**Table 4:** 3D MPRAGE acquisition parameters for each relevant site in the ABIDE dataset (Kucharsky Hiess et al., 2015). The scanner used at all sites was a Siemens Magnetom.

Site	Controls (m/f)	ASD (m/f)	Image ac- quisition	Voxel size (*mm*^3^)	Flip angle (deg)	TR (ms)	TE (ms)	TI (ms)	BW (Hz/ Px)
CALTECH^a^	15/4	15/4	3D MPRAGE	1×1×1	10	1590	2.73	800	200
CMU^b^	10/3	11/3	3D MPRAGE	1×1×1	8	1870	2.48	1100	170
NYU^c^	79/26	68/11	3D MPRAGE	1.3×1×1.3	7	2530	3.25	1100	200
OLIN^d^	13/3	18/2	3D MPRAGE	1×1×1	8	2500	2.74	900	190
OHSU^e^	15/0	15/0	3D MPRAGE	1×1×1	10	2300	3.58	900	180
${\text{UCLA}}_{\text{1}}^{f}$	29/4	42/7	3D MPRAGE	1×1×1.2	9	2300	2.84	853	240
${\text{UCLA}}_{\text{1}}^{g}$	12/2	13/0	3D MPRAGE	1×1×1.2	9	2300	2.84	853	240
PITT^h^	23/4	26/4	3D MPRAGE	1.1×1.1×1.1	7	2100	3.93	1000	130
USM^i^	43/0	58/0	3D MPRAGE	1×1×1.2	9	2300	2.91	900	240
YALE ^j^	20/8	20/8	3D MPRAGE	1×1×1	9	1230	1.73	624	320

a California Institute of Technology

b Carnegie Mellon University

c NYU Langone Medical Center, New York

d Olin, Institute of Living, Hartford Hospital

e Oregon Health and Science University

f,g University of California, Los Angeles

h University of Pittsburgh School of Medicine

i University of Utah School of Medicine

j Child Study Centre, Yale University

**Table 5:** Trends statistics across sites, per age-partitioned group, tissue type, and experiment. Standard deviations are quoted in brackets. Bold values denote improvement over SPM-C.

	Grey Matter				White Matter				CSF			
Experiments	”Young”		”Old”		”Young”		”Old”		”Young”		”Old”	
	b	m (x10^−3^)	b	m (x10^−3^)	b	m (x10^−3^)	b	m (x10^−3^)	b	m (x10^−3^)	b	m (x10^−3^)
SPM (Baseline)	0.6261 (0.0338)	-5.0864 (3.1716)	0.5677 (0.0465)	-2.2552 (1.2661)	0.2754 (0.0121)	0.7202 (0.9218)	0.2746 (0.0190)	1.0360 (0.7280)	0.0985 (0.0442)	4.3661 (3.9279)	0.1577 (0.0653)	1.2192 (1.9581)
CNN Baseline	0.5584 (0.0382)	-5.9047 **(0.8932)**	0.4435 (0.0773)	-2.0377 (1.2051)	0.3848 (0.0236)	0.3095 (0.7452)	0.4481 (0.0972)	0.9013 (0.3833)	0.0569 **(0.0230)**	5.5952 (1.3503)	0.1084 (0.0340)	1.1364 (1.3545)
PSACNN	0.4457 **(0.0080)**	-0.5686 **(0.6376)**	0.4540 **(0.0053)**	-0.8821 **(0.2807)**	0.4487 (0.0148)	-0.3971 (0.5993)	0.4306 (0.0111)	0.0636 **(0.2355)**	0.1056 **(0.0099)**	0.9657 **(0.6027)**	0.1154 **(0.0095)**	0.8184 **(0.3920)**
SynthSeg	0.5287 **(0.0081)**	-1.8472 **(0.5637)**	0.5031 **(0.0098)**	-0.9001 **(0.0959)**	0.3196 (0.0117)	2.0294 (0.8456)	0.3430 (0.0112)	0.5249 (0.3229)	0.1517 **(0.0090)**	-0.1823 **(0.7813)**	0.1539 **(0.0272)**	0.3752 **(0.3141)**
Strat-Aug	0.5353 **(0.0144)**	-3.7060 **(0.8859)**	0.5000 (0.0174)	-1.8534 **(0.4316)**	0.3736 (0.0208)	1.0884 (0.7026)	0.3923 (0.0227)	0.6958 **(0.2525)**	0.0911 **(0.0254)**	2.6176 **(1.1613)**	0.1077 (0.0388)	1.1575 **(0.5582)**
Phys-Strat-Aug	0.5475 **(0.0114)**	-3.8187 **(0.7150)**	0.4962 (0.0265)	-1.7860 **(0.3925)**	0.4048 (0.0210)	1.2285 (0.5688)	0.4236 (0.0237)	0.7272 **(0.2777)**	0.0477 **(0.0179)**	2.5902 **(0.5908)**	0.0802 **(0.0244)**	1.0587 **(0.5326)**
SPM-C	0.6286 (0.0451)	-4.5689 (3.9178)	0.5691 (0.0340)	-2.1296 (1.3734)	0.2797 (0.0107)	0.7158 (0.8418)	0.2716 (0.0195)	1.0877 (0.7771)	0.0947 (0.0541)	3.5818 (4.4873)	0.1554 (0.0463)	1.1982 (1.8874)
CNN Baseline-C	0.5590 (0.0384)	-6.0404 **(1.0361)**	0.4574 (0.0320)	-2.0423 (1.1976)	0.3799 (0.0097)	0.2363 (0.8568)	0.4145 (0.0538)	0.9936 (0.5009)	0.0609 **(0.0114)**	5.8040 (1.4680)	0.1336 (0.0345)	0.8382 (1.3743)
PSACNN-C	0.4437 **(0.0088)**	-0.4821 **(0.7567)**	0.4507 **(0.0086)**	-0.8173 **(0.3490)**	0.4495 (0.0086)	-0.3945 (0.6048)	0.4318 (0.0121)	0.0669 **(0.2338)**	0.1055 **(0.0087)**	0.9697 **(0.6214)**	0.1166 **(0.0073)**	0.7835 **(0.3024)**
SynthSeg-C	0.5290 **(0.0082)**	-1.8312 **(0.6192)**	0.5068 **(0.0022)**	-0.8958 **(0.0981)**	0.3222 (0.0209)	2.0446 (0.8630)	0.3459 (0.107)	0.5569 (0.4041)	0.1466 **(0.0079)**	-0.0268 **(0.5980)**	0.1488 **(0.0310)**	0.2881 **(0.4341)**
Strat-Aug-C	0.5333 **(0.0132)**	-3.7094 **(0.8908)**	0.5012 (0.0133)	-1.8832 **(0.4345)**	0.3738 (0.0201)	1.0993 (0.7404)	0.3885 (0.0231)	0.7313 **(0.2887)**	0.0932 **(0.0248)**	2.5794 **(1.0293)**	0.1098 **(0.0294)**	1.1668 **(0.5296)**
Phys-Strat-Aug-C	0.5525 **(0.0111)**	-3.8440 **(0.8408)**	0.5006 **(0.0105)**	-1.7847 **(0.4032)**	0.3967 (0.0138)	1.2319 **(0.4466)**	0.4212 (0.0162)	0.7639 **(0.2978)**	0.0506 **(0.0142)**	2.6277 **(0.6620)**	0.0783 **(0.0216)**	1.0160 **(0.5235)**

## Data Availability

The YOAD was acquired at the National Hospital for Neurology and Neurosurgery, Queen Square. This dataset is not openly available. Further details regarding the ABIDE data employed by this work, and the means to gain access to it, can be sought via http://fcon_1000.projects.nitrc.org/indi/abide/. Gaining access requires users to register with the NITRC and 1000 Functional Connectomes. Data used in this article are partly from the Alzheimer’s Disease Neuroimaging Initiative database (http://adni.loni.usc.edu). Investigators in the ADNI contributed to the design and implementation of ADNI and/or provided data but did not participate in analysis of this report. A complete listing of investigators at: adni.loni.usc.edu/wpcontent/ADNI Acknowledgement List.pdf Code supporting the findings of this work, as well as a means to run some of our physics-informed models can be found in the following GitHub repository (https://github.com/pedrob37/Phys_Seg) under a permissive OpenSource license.

## Footnotes

1 The ADNI was launched in 2003 as a public-private partnership, led by Principal Investigator Michael W. Weiner, MD. The primary goal of ADNI has been to test whether serial magnetic resonance imaging (MRI), positron emission tomography (PET), other biological markers, and clinical and neuropsychological assessment can be combined to measure the progression of mild cognitive impairment (MCI) and early Alzheimer’s disease (AD). For up-to-date information, see www.adni-info.org.
