# Supplementary material for: Acquisition-invariant brain MRI segmentation with informative uncertainties
Source: Med Image Anal. Author manuscript; Available in PMC 2024 Dec 11. (PMC7617170; doi:10.1016/j.media.2023.103058)
Supplement: Appendix A [file EMS198236-supplement-Appendix_A.pdf]

Supplementary Materials

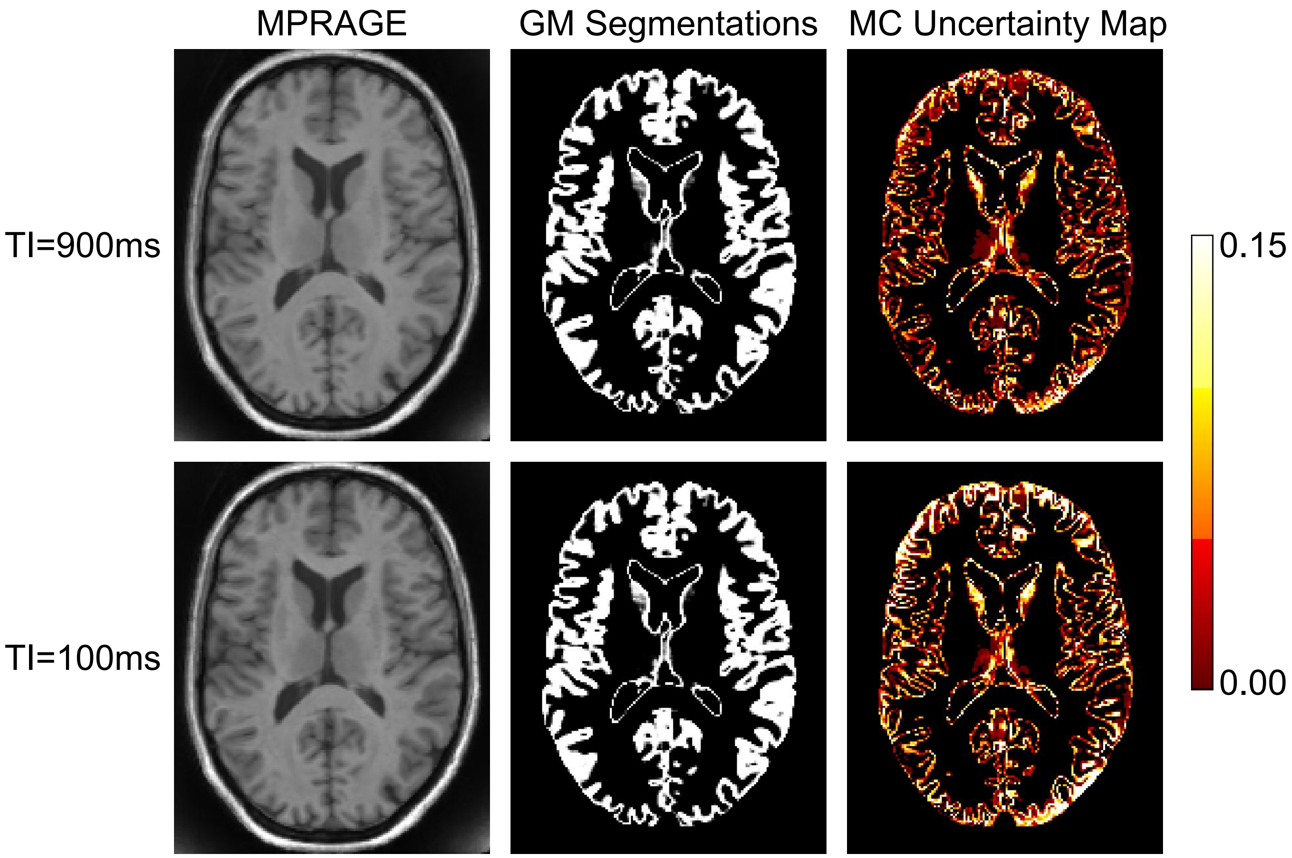

Fig. 1: Uncertainty-aware *Phys-Strat-Aug* grey matter segmentations for shown simulated MPRAGE axial slices for inference subject with accompanying Monte Carlo standard deviation uncertainty maps. Note how despite the segmentations being virtually indistinguishable, a greater uncertainty is observed in the out of distribution map.

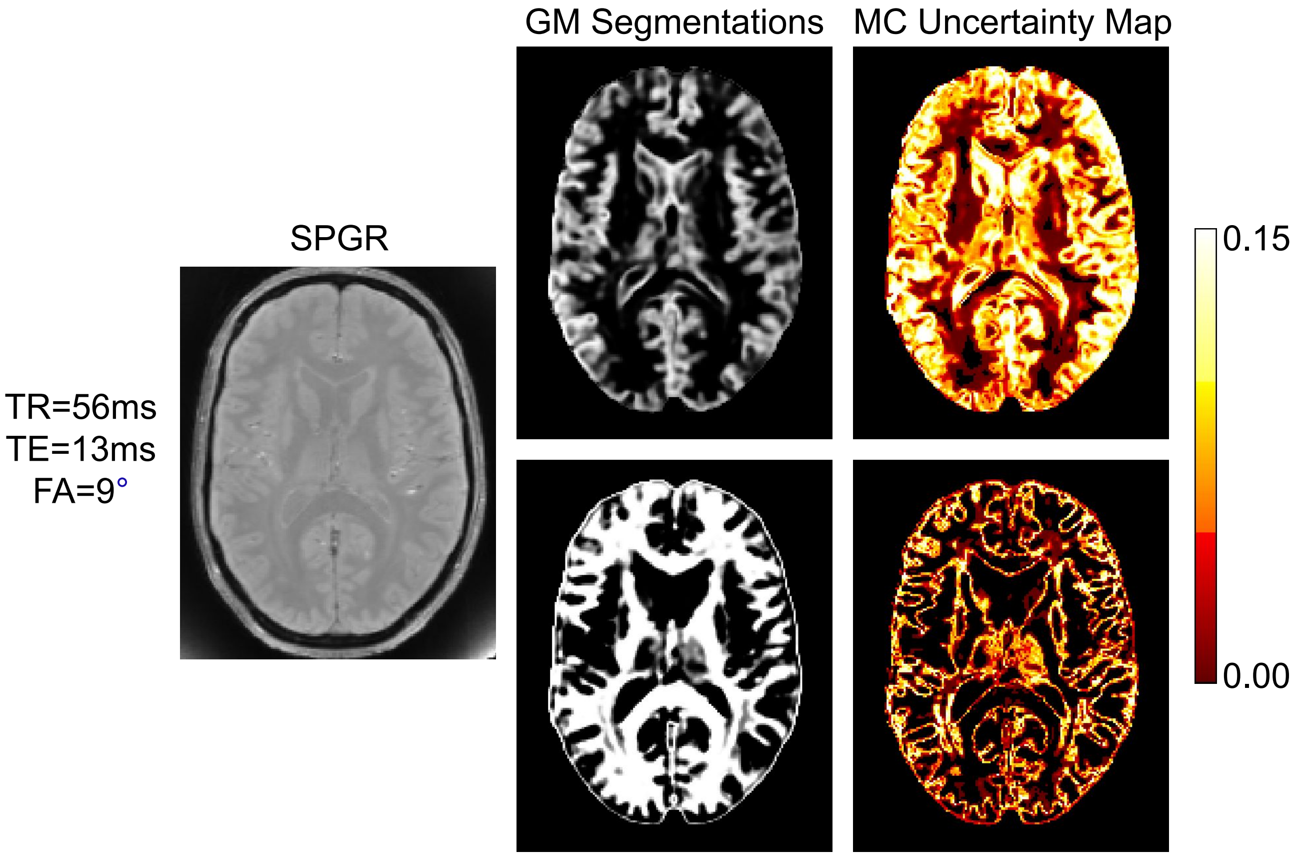

Fig. 2: Uncertainty-aware *Phys-Strat-Aug* (Top) and uncertainty-aware *Baseline* (Bottom) grey matter segmentations for shown simulated out of distribution SPGR axial slice for inference subject with accompanying Monte Carlo standard deviation uncertainty maps. Neither method produces a good quality segmentation, but *Phys-Strat-Aug*'s uncertainty map exhibits a much higher uncertainty than the Baseline model.

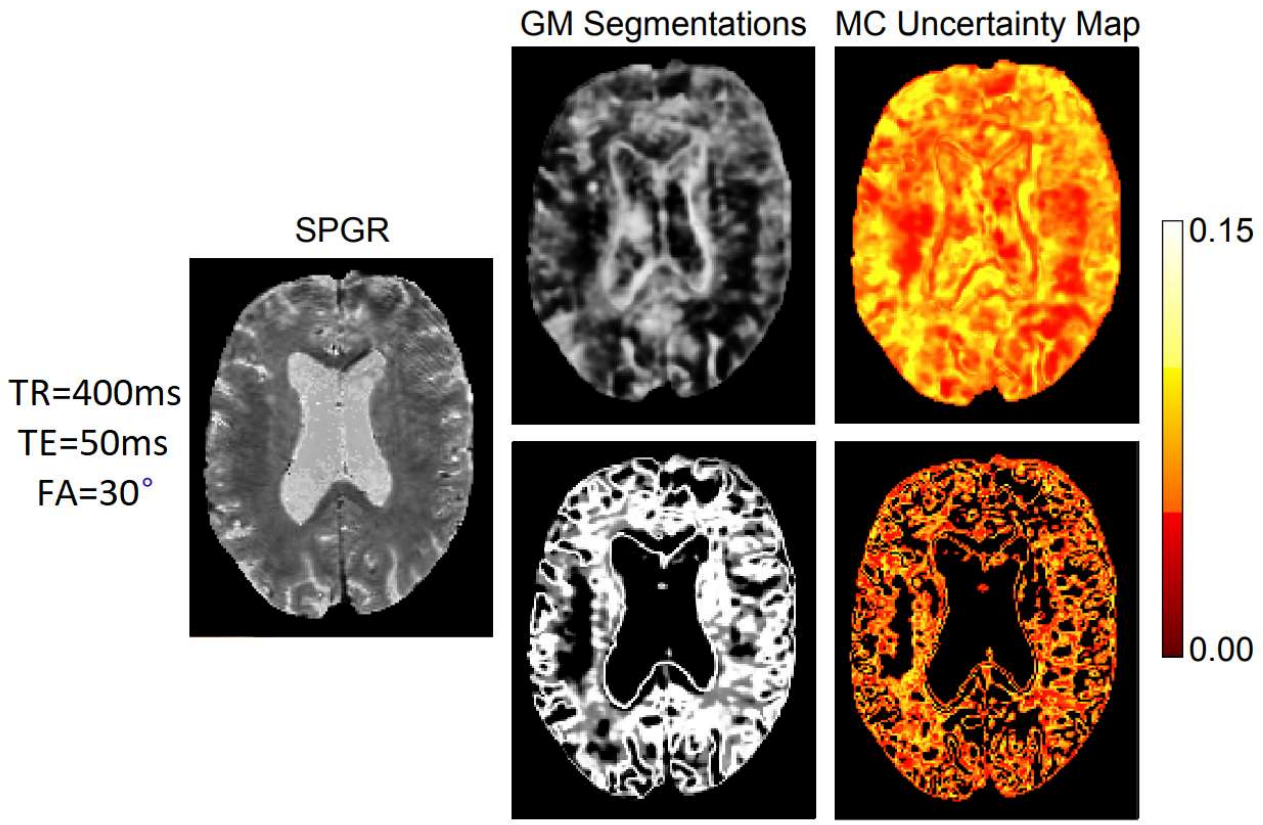

Fig. 3: Uncertainty-aware *Phys-Strat-Aug* (Top) and uncertainty-aware *Baseline* (Bottom) grey matter segmentations for shown simulated out of distribution (High TR and high TE) SPGR axial slice for inference subject with accompanying Monte Carlo standard deviation uncertainty maps. Neither segmentation is viable, but *Phys-Strat-Aug*'s uncertainty map exhibits a much higher uncertainty than the *Baseline* model.

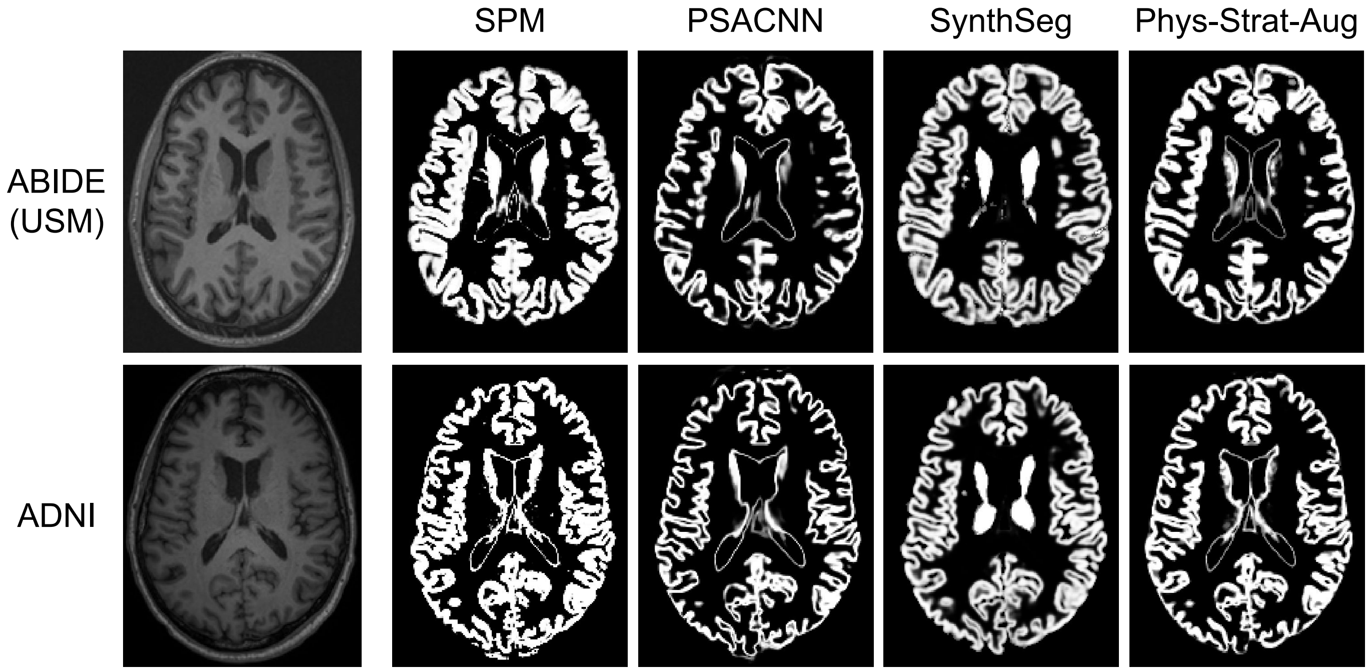

Fig. 4: Grey matter segmentations from *SPM*, *PSACNN*, *SynthSeg*, and *Phys-Strat-Aug*, for example ABIDE (USM site) and ADNI subjets.
